# Robot-assisted line bisection in patients with Complex Regional Pain Syndrome

**DOI:** 10.1371/journal.pone.0213732

**Published:** 2019-05-02

**Authors:** Charlotte Verfaille, Lieve Filbrich, David Cordova Bulens, Philippe Lefèvre, Anne Berquin, Olivier Barbier, Xavier Libouton, Virginie Fraselle, Dominique Mouraux, Valéry Legrain

**Affiliations:** 1 Institute of Neuroscience, Université catholique de Louvain, Brussels, Belgium; 2 Psychological Sciences Research Institute, Université catholique de Louvain, Louvain-la-Neuve, Belgium; 3 Institute of Information and Communication Technologies, Electronics and Applied Mathematics, Louvain-la-Neuve, Belgium; 4 Saint-Luc University Hospital, Brussels, Belgium; 5 Institute of Experimental and Clinical Research, Université catholique de Louvain, Brussels, Belgium; 6 Faculty of Motor Sciences, Université catholique de Louvain, Louvain-la-Neuve, Belgium; 7 Faculty of Motor Sciences, Université libre de Bruxelles, Brussels, Belgium; 8 Erasme University Hospital, Brussels, Belgium; Washington University in Saint Louis School of Medicine, UNITED STATES

## Abstract

Complex Regional Pain Syndrome (CRPS) is characterized by pain, motor and inflammatory symptoms usually affecting one limb. Cognitive difficulties have been reported to affect patients’ ability to represent, perceive and use their affected limb. It is debated whether these difficulties result from deficits in controlling goal-directed movements in space or from a learned strategy to protect the affected limb. In order to dissociate the two hypotheses, patients with upper-limb CRPS were asked to move with their unaffected hand towards visual targets projected at different positions on a horizontal semi-reflexive mirror. By means of a robotic handle placed below the screen, they were asked to move a cursor, to reach and cross lines at their estimated midpoint. In some of the stimulation series, the affected hand was placed below the mirror so that some lines appeared projected onto that hand. Vision of the hands and the robotic handle was preserved or prevented by opening or closing a shutter below the mirror. Lines were displayed on the mirror according to which part of the body was affected (ispi- vs. contralateral) and the actual position of the affected hand (inside vs. outside the workspace). Comparatively to control participants, CRPS patients generally biased their estimation by bisecting the lines towards their left side, irrelative of which part of the body was affected and the position of the affected hand, both in ipsi- and contralateral space, with only a few exceptions. Our results are in line with previous studies having described a visuospatial deficit in CRPS patients and discard the explanation of observed symptoms in terms of learned nonuse strategies, as only the unaffected hand was used to perform the task. It is suggested that CRPS patients can display difficulties to perform tasks requesting visuo-motor coordination, reflecting the complex cortical reorganization occurring in CRPS.

## Introduction

Complex Regional Pain Syndrome (CRPS) is a chronic pain condition usually affecting one limb that is characterized by sensory, sudomotor and vasomotor symptoms (e.g. pain, edema, temperature changes or trophic changes) [1, 2]. The affected limb has also been clinically reported to be afflicted by motor symptoms such as a lack of spontaneous movement, underuse (e.g. most of the actions are performed with the opposite healthy limb), hypokinesia, bradykinesia and limited range of motion [3–5]. Additionally, patients suffering from CRPS have been described as presenting impaired abilities to represent, perceive and move their affected limb [6–8], supporting the hypothesis of a cortical contribution of the pathophysiology of CRPS [1]. For instance, CRPS patients described their affected limb as feeling disconnected from their body as well as having trouble initiating movements and performing actions with it [3]. These observations have led some authors to suggest that cognitive symptoms in CRPS might be similar to the symptomatology observed in hemispatial neglect (HSN) [3], a cognitive disorder consecutive to a brain lesion characterized by a deficit in perceiving and exploring sensory events from the side of space contralateral to the lesion [9, 10].

The exact nature of the neglect-like symptomatology in CRPS is still a matter of debate [6, 11], but this issue has potential clinical implications as it was suggested that pain and other CRPS-related symptoms might be alleviated by rehabilitation techniques used to treat perceptual and motor symptoms in HSN (e.g. [12, 13, 14]). However, HSN is often, if not exclusively, characterized and diagnosed based on symptoms affecting patients’ visuospatial perceptual abilities. Neuropsychological tests classically used to investigate visuospatial perception in HSN failed to evidence the presence of symptoms of visuospatial neglect in CRPS [15, 16]. Indeed, visuospatial deficits were only highlighted in CRPS patients by means of very specific laboratory situations such as temporal order judgement (TOJ) tasks, a psychophysical testing during which participants have to report which of two sensory events, presented in succession with quite small time asynchrony, is perceived as first occurred [17, 18]. Another important issue regarding the comparison between CRPS and HSN is that, in HSN, the side of space affected by disrupted perception is defined according to which cortical hemisphere is damaged, whereas in CRPS it usually depends on the affected side of the body [17–19]. For these reasons, some authors argued that perceptual deficits in CRPS could be too subtle to be clinically relevant, while, in turn, motor deficits of the affected limb might reflect learned strategies to avoid provocation of the affected limb instead of HSN symptomatology [11]. Indeed, according to the learned nonuse hypothesis, consecutively to the initial injury or trauma, patients might learn to avoid using the affected limb, due to pain, restricted movement capacities or enforced immobility, and develop strategies to compensate the underuse of the affected limb by overusing the unaffected one (for a review, see [20]). By contrast, HSN symptomatology cannot be entirely explained by deficits to primary motor function, so that motor deficits observed in HSN are not only characterized by an inability to use the limb contralateral to the side of the lesion, but also by difficulties in initiating and executing goal-directed movements, with both hands, in the direction of the contralesional side of space [21–23]. Accordingly, the objective of the present study was to investigate in CRPS patients the existence of directional motor difficulties dissociated from learned nonuse strategy, by investigating the abilities of CRPS patients to move and reach a target with their unaffected limb. To dissociate between these two concepts, we adapted the line bisection task, a classic neuropsychological test of HSN that consists on reaching and pointing to the midpoint of horizontal lines (performance of HSN patients is usually characterized by bisection biases towards the preserved side of space) [24, 25], using a robotic-assisted virtual reality system. In brief, lines were presented by means of a horizontal mirror placed in front of patients suffering from upper-limb CRPS, and they were instructed to move a visual cursor towards the estimated midpoint of each line by holding and moving the handle of the robotic device with their unaffected hand. In comparison to standard paper-and-pencil tasks, this device allowed us to precisely control the presentation of the visual stimuli and finely analyze participants’ pointing movements [26]. We hypothesized that, according to the learned nonuse hypothesis, movements of the unaffected limb should not be hindered during visuo-motor coordination task, and therefore, patients with CRPS should not perform the task differently than control participants. Conversely, if CRPS patients present cognitive deficits similar to those observed in HSN, movements with the healthy limb could be hindered, especially when directed towards lines projected in the side of the pathological limb, resulting in bisection errors. However, because comparing CRPS to a syndrome involving cortical lesions can be difficult and due to the great results variability observed in previous studies with CRPS patients [15, 16, 27–30], we decided not to make any assumptions as to the spatial direction of the bisection errors. It is also worth noting that in case we show such bisection biases in CRPS patients, our design does not allow dissociating between a perceptual or motor origin of the observed cognitive symptoms.

In addition, we also investigated whether CRPS could affect differently and specifically some spatial frames of reference that correspond to different coordinate systems that the brain uses to process the spatial location of sensory stimuli (for a review, see [31]). For instance, Filbrich et al. [17] suggest that visuospatial difficulties of CRPS patients could affect more particularly their ability to perceive visual stimuli presented near the affected hand, than those presented in the same side of space but at a farther distance from the affected hand. Accordingly, we investigated whether possible difficulties of CRPS patients to execute spatially directed movements could affect more specifically their ability to move and reach visual stimuli presented in the space immediately surrounding the affected hand, as compared to stimuli still presented in the side of space corresponding to the pathological part of the body but at a farther distance from the hand. In the present experiment, lines were presented in both sides of space, contralateral and ipsilateral to the affected hand, and some lines were presented right above the affected hand or beside it. Different procedures were used to test whether patients’ performance was dependent on the presence of the hand in the workspace of the lines, as driven by proprioception and/or its direct vision. We hypothesized that, in the frame of the learned nonuse hypothesis, CRPS patients should not have difficulties to move their unaffected hand towards any lines. On the contrary, if CRPS patients have difficulties similar to those observed in HSN to perform spatially-directed movements with their unaffected hand, and if such difficulties specifically affect movements towards the space around the affected hand, bisection biases should be particularly observed for lines presented directly in the close vicinity of the affected hand.

## Methods

### Participants

**Fig 1:**
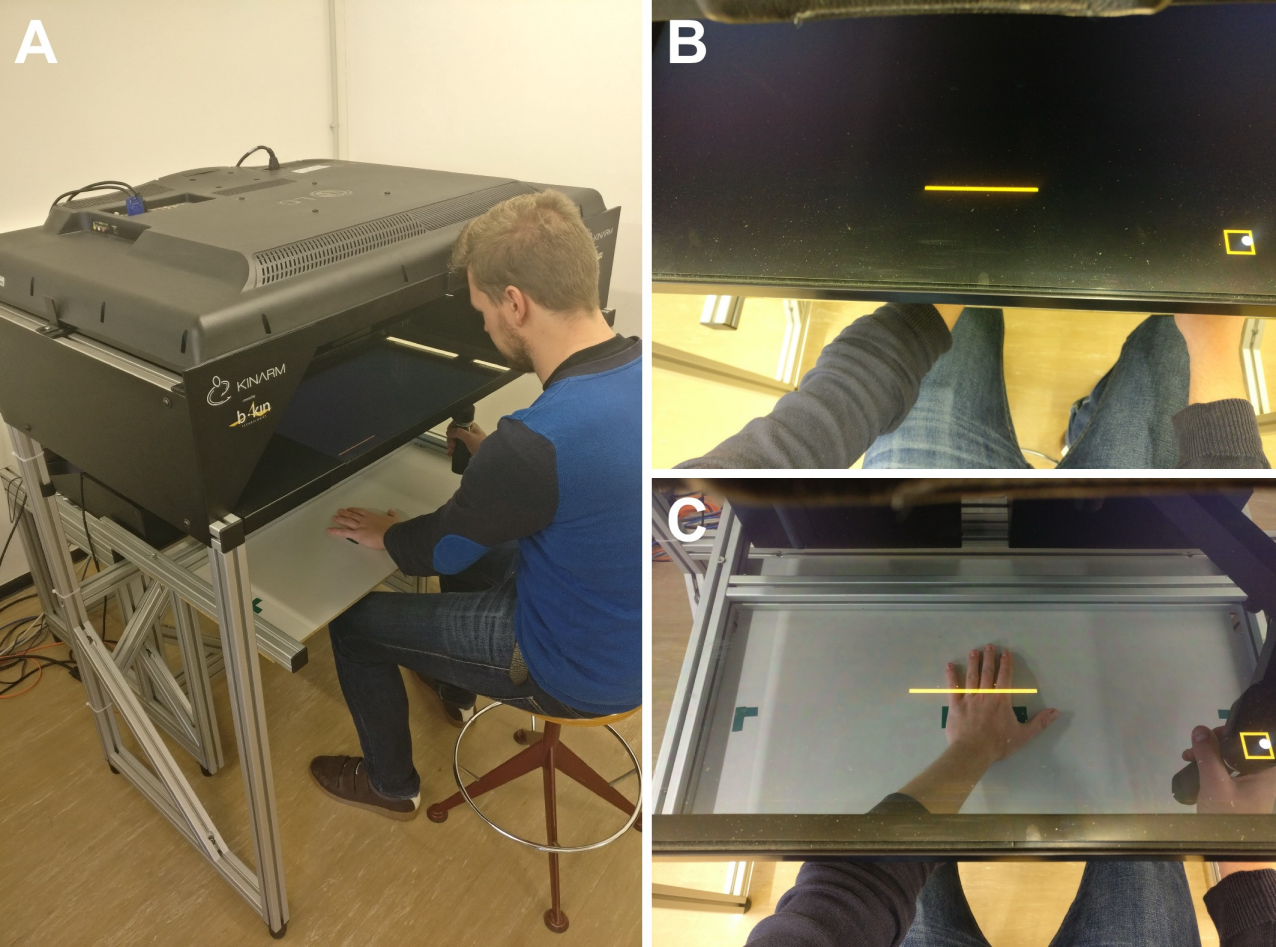
Picture of the KINARM robotic device. (A) The KINARM is a robotic laboratory device designed to study visuo-motor coordination and is made of two systems. First, the robotic section made of two graspable handles allows encoding various movement-related parameters such as trajectory and positions. Second, just above the robotic section, the virtual reality system is made of a horizontal semi-reflective mirrored screen. This device allows combining direct observation of the robotic section and reflection of visual stimuli projected from a monitor at the top of the device. It also allows creating a virtual reality environment in which visual stimuli are perceived as occurring in the same space of the limb. (B) The direct vision of the hand can be occluded through the semi-reflective screen by closing two sliding shutters. Once closed, only visual stimuli and the reach-to-point cursor can be seen. (C) While open, the hands as well as the experimental setting are visible through the screen. The person in the photograph is a member of the laboratory who served as a witness to illustrate the experimental setup and gave his consent to publish this picture.

Sixteen patients with upper-limb CRPS were recruited in collaboration with the orthopedic and physical rehabilitation departments of Saint-Luc University Hospital (Brussels, Belgium), Erasme University Hospital (Brussels, Belgium) and Bois de la Pierre Clinical Center (Wavre, Belgium) between May 2015 and July 2016. The testing took place at Université catholique de Louvain (Brussels, Belgium). One patient was removed from the analyses because of incomplete testing due to discomfort related to pain. Twelve participants fulfilled the Budapest clinical criteria for CRPS, of whom five also fulfilled the Budapest criteria for research [2]. Three additional participants were diagnosed according to non-specified criteria by orthopedic surgeons based on repeated clinical examinations and corroborated by bone scintigraphy [32]. Exclusion criteria were any neurological and severe psychiatric disorders, unresolved orthopedic injuries as well as uncorrected impaired vision. The mean age of the CRPS participants (10 women) was 49 years (SD = 8.5, range: 37–65 years). Nine of them were affected by a CRPS on the left hand while 6 presented a CRPS on the right hand. According to the Flinders Handedness Survey [33], all participants were right-handed, except one who was left-handed (see details in Table 1). Fifteen healthy volunteers were recruited as control participants and were individually matched to each of the CRPS patients according to age (M = 49, SD = 8.5, range: 37–65 years), gender and handedness. The recruitment and experimental procedures of this study were approved by the local ethic committee (Commission d’Ethique Biomédicale Hospitalo-Facultaire des Cliniques universitaires Saint-Luc et de l’Université catholique de Louvain) and conform to the Declaration of Helsinki. All participants provided written informed consent before taking part in the study and received a financial compensation for their participation. The individual in Fig 1 has given written informed consent (as outlined in PLOS consent form) to publish these case details.

**Table 1:** Characteristics of the CRPS participants. # = patient number; Age in years; Hand = handedness; F = female; M = male; R = right; L = left; STI = soft tissue injury; Frac = fracture, PS = post-surgery; *CE* = clinical examination; CRPS-R = Budapest research criteria for CRPS; CRPS-C = Budapest clinical criteria for CRPS; II = CRPS due to nerve injury; Dur = duration since inciting injury in months; PT = physical therapy; OT = occupational therapy.

#	Age/sex/hand.	Inciting injury	CRPS limb	Diagnosis	Dur	Current treatment/medication	Other pain	Other
01	37/F/R	STI wrist	L	*CE*	15	PT	/	/
02	47/F/R	STI wrist	L	CRPS-R	32	PT/OT/Amitriptyline (10mg /1 per day)	/	Glau-coma
03	44/M/R	Frac thumb	L	*CE*	5	PT	/	/
04	46/M/R	Frac-PS wrist	R	*CE*	3	PT	/	/
05	65/F/R	Frac-PS wrist	L	CRPS-C	6	PT/Paracetamol, Ibuprofen (when on pain)	/	/
06	52/F/R	STI cut nerve-PS	L	CRPS-C II	3	PT/Paracetamol (1g/ 2 or 3 per day),Pregabalin (150mg / 2 per day)	/	/
07	49/M/R	Frac fingers	R	CRPS-C	2.5	PT	/	/
08	38/F/R	STI wrist	R	CRPS-C	6	PT	L hand, R ankle	/
09	54/F/L	Frac wrist	L	CRPS-R	4	PT	R shoulder	/
10	38/M/R	Frac wrist	R	CRPS-R	9	PT	/	/
11	47/F/R	PS wrist	R	CRPS-C	18.5	PT/OT/Pregabalin (150mg / 3 per day), Topiramate (15mg / 2 per day), Duloxetine (60mg / 1 per day)	/	/
12	48/F/R	STI hand	L	CRPS-R	17	PT/OT/Amitriptyline (10mg / 1 per day)	/	/
13	61/F/R	Frac-PS wrist	L	CRPS-R	6	PT/OT/Tramadol (when on pain)	L foot	/
14	54/F/R	PS hand	R	CRPS-C	10	PT/OT/Tramadol (150mg / 2 per day)	/	/
15	60/M/R	Frac-PS wrist	L	CRPS-C	9.5	PT	/	/

### Stimuli & apparatus

**Fig 2:**
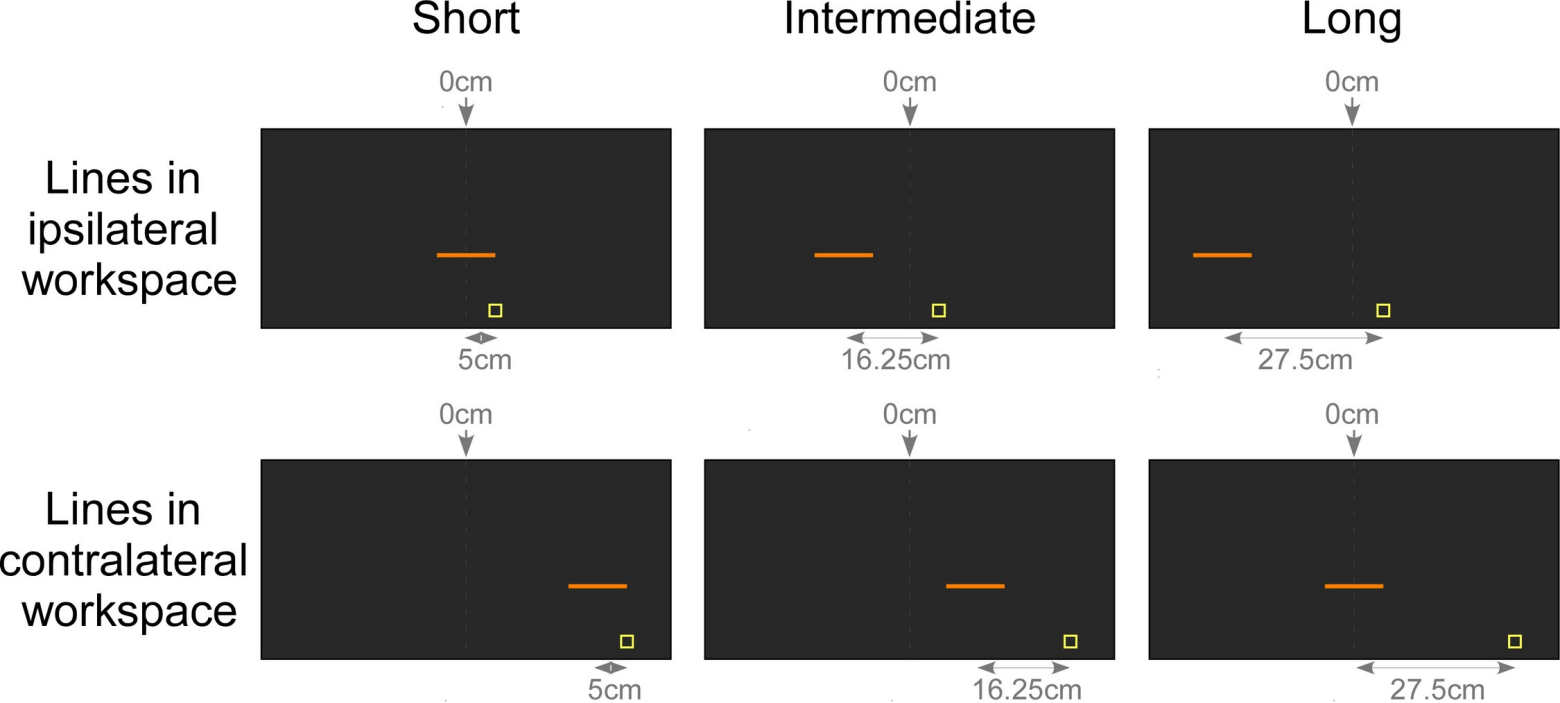
Display of lines projected in the KINARM. The figure illustrates conditions during which participants performed the task with the right hand, while the left hand, corresponding to the CRPS hand in this example, remained static. In different blocks, the lines were presented either in the workspace ipsilateral to the static hand (upper part) or in the contralateral workspace (lower part). Dash lines at 0 cm correspond to the vertical median of the semi-reflexive screen to which participant’s body midline was aligned. The lines were project at three different distances: the midpoint of the line was positioned at 0 cm, 11.25 cm or 22.50 cm relatively to the vertical median of the mirror. For experimental purposes, the exact position of the lines was computed relative to the starting position of the movement cued by the yellow square. When working in the space ipsilateral to the static hand the yellow square appeared at a distance of 5 cm laterally from the median of the mirror in the direction of the opposite workspace. When working in the contralateral space, the yellow square appeared at a distance of 27.5 cm laterally from the median of the mirror in the same workspace. As a consequence, lines were presented at 5 cm from the starting position (short distance, left part), at 16.25 cm (intermediate distance, middle part), or at 27.5 cm (long distance, right part).

During the experiment, participants were asked to bisect lines, projected at different space positions on a horizontal screen, at their midpoint. To this aim, we used the KINARM robotic system (BKIN Technologies, Kingston, ON, Canada) to conduct this experiment. This device is made up of two graspable robotic handles and a horizontal semi-reflective mirror (36 cm wide x 69.5 cm long) placed above the handles (Fig 1). Participants controlled one of the robotic handles to move a cursor towards visual targets displayed on the semi-reflexive mirror by means of a horizontal 16-to-9 52-inches monitor (75 Hz refresh rate) placed above the device. The cursor was a 7-mm diameter plain white circle spatially locked to the location of the handle midpoint of the active robot. Visual targets were orange lines of three different lengths: 10, 15 and 20 cm width (0.3 cm of thickness) projected perpendicularly to the participants’ sagittal plane on the semi-reflective mirror. The lines were presented at a distance of 15 cm in depth on the antero-posterior axis with regard to the proximal edge of the mirror, and at three different lateral positions (i.e. on the transversal axis) so that the midpoint of the line was positioned at 0 cm, ±11.25 cm or ±22.50 cm relatively to the vertical median of the mirror (negative values correspond to positions in the left hemispace of the mirror, positive values to positions in the right hemispace). During each block, the task was realized with the lines projected in only one hemispace, that is, in one part of the mirror (0, -11.25 and -22.50 cm in the left hemispace or 0, 11.25, 22.50 cm in the right hemispace) (Fig 2).

### Procedure

**Fig 3:**
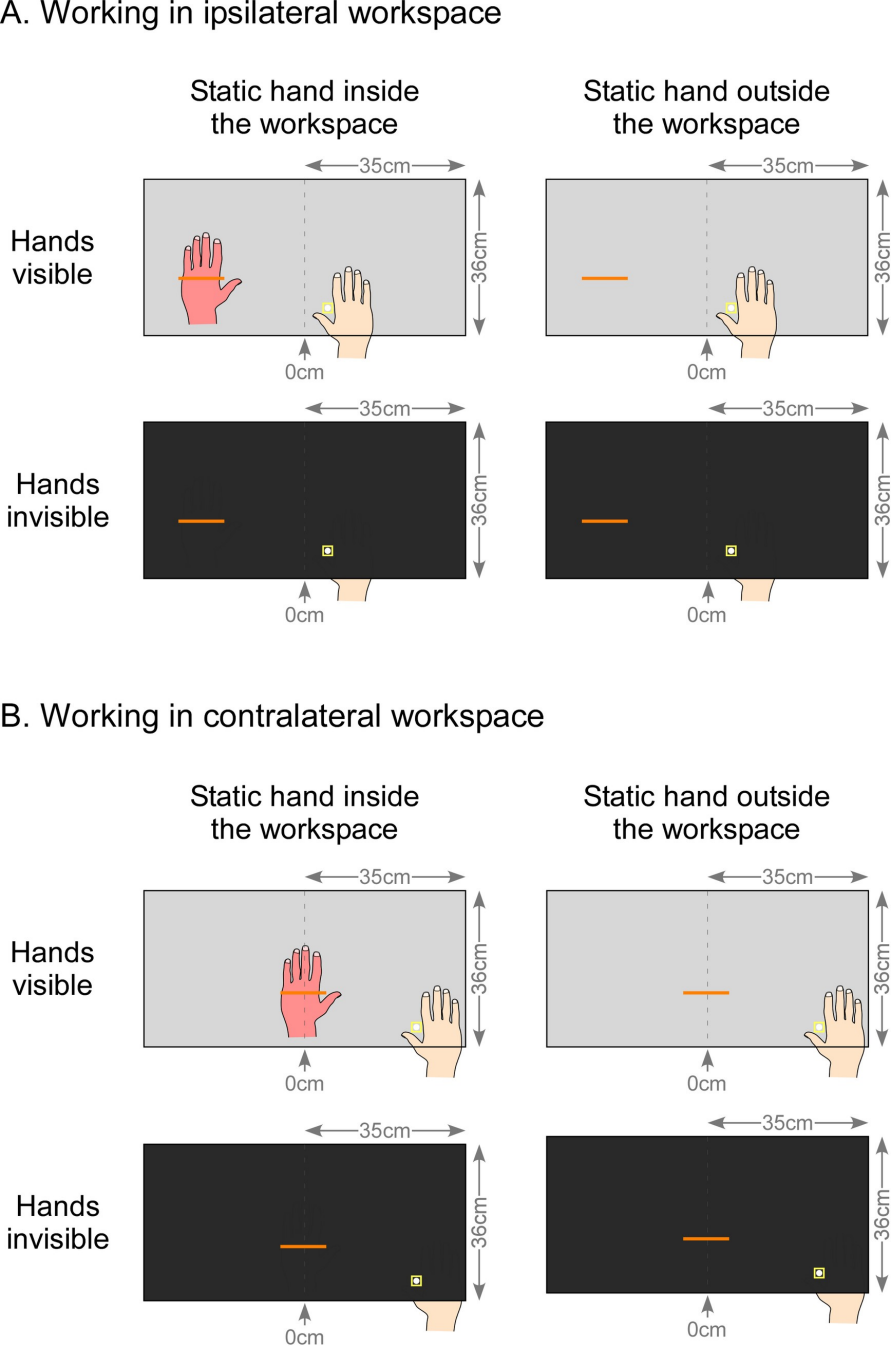
Experimental conditions. The figure illustrates the line bisection task using the KINARM performed by a hypothetical participant with a left static hand. Dash lines at 0 cm correspond to the vertical median of the semi-reflexive screen to which participant’s body midline was aligned. During the task, line bisections were presented in the workspace according to two conditions: in half the task, lines were projected in the workspace ipsilateral to the static hand position (A), while in the other half, they were displayed in the contralateral workspace (B). Note that the figure only depicts the lines aligned with the static hand when placed inside the workspace. Indeed, the static hand was placed either inside the workspace (left part of the figure) or it was kept outside the workspace in a resting position, close to the trunk (right part). Finally, participants were either able to see their hands and the experimental setting through the semi-reflexive mirror (upper part of panels A and B) or they could not see through the screen (lower part of each panel).

Participants sat in front of the screen in order to match their body midline with the vertical median of the mirror. Their forehead rested on a specific support to align the head with the center of the workspace, but head and gaze movements were not constrained (see Fig 1A). During the task, for both the CRPS and control participants, projection and bisection of the lines were manipulated according to 5 variables: which hand is used to perform the task, the workspace in which the lines appeared, the possibility to see the hands during the task, the position of the hand that remained static, and the distance of the lines relatively to the starting position of the cursor (Figs 2 & 3). During the entire experiment, only the unaffected hand of the CRPS participants was used as active while the affected one rested static. Similarly, each control participant performed the task with only one active hand which was the same hand as the CRPS participant to which they were matched, independently of their handedness (w*hich is used*). In order to investigate spatially-guided visuo-motor coordination according to the HSN hypothesis, the lines were projected in the hemispace of the mirror ipsilateral to the static hand during one half of the experimental blocks (*workspace*: *ipsilateral condition*), and in the hemispace contralateral to the static hand during the other half of the blocks (*workspace*: *contralateral condition*) (Fig 3). Next, hands and workspaces were made either visible though the mirror (*vision of the hands*: *visible condition*) or invisible by closing the shutters below the mirror and occulting any vision of the hands and the robot handle (*vision of the hands*: *invisible condition*). This was intended to test whether potential visuo-motor deficits in CRPS were driven by visual inputs (seeing the hand) or proprioceptive inputs (feeling its position) when vision was prevented. Additionally, to test whether deficits were spatially locked to the affected hand’s position, the static hand of both CRPS and control participants was placed according to two positions. This latter was either kept close to the trunk (*position of the static hand*: *outside workspace condition*) or placed in the workspace (*position of the static hand*: *inside workspace condition*). The exact position of the static hand inside the workspace was dependent of the hemispace in which lines were projected (i.e. *ipsilateral* vs. *contralateral*). In the *ipsilateral condition*, the static hand was spatially aligned with the line located at the most lateral distance, i.e. the ±22.5-cm line. In the *contralateral condition*, the static hand was spatially aligned with the line located at the most median distance, on the body midline, i.e. the 0-cm line. More precisely, the articulation between the metacarpus and the proximal phalanx of the little finger of the static hand was aligned with a mark on the table located at 8 cm horizontally (more laterally) and at 2 cm vertically (more deeply) from the midpoint of the respective reference line (Fig 3).

CRPS and control participants were presented with 8 blocks resulting from the conditions *workspace* (ispi- vs. contralateral), *vision of the hands* (visible vs. invisible) and *position of the static hand* (inside vs. outside). Within each block, lines were of 3 different lengths (10, 15, 20 cm) and distances from a starting position (0, ±11.25, ±22.50 cm). Each type of line was repeated 10 times, resulting in 90 trials per block. Blocks were balanced according to the *position of the static hand* conditions and randomly presented according to the other variables. Before the beginning of each block, participants were asked to grasp one handle of the robot with their active hand. Each trial started with the appearance of a yellow square (2*2 cm) at a 5-cm distance in depth with regard to the proximal edge of the mirror to cue the starting position of the hand movement: participants were instructed to put the cursor into the yellow square in order to start the task. Once in position, a line was displayed on the screen and they were asked to move the cursor in the direction of the line in order to bisect it at its estimated midpoint as accurately and smoothly as possible. Participants were asked to adopt their natural speed, not too slow or fast. The yellow square disappeared when the cursor was moved out of it and once the line was crossed by the cursor, the line disappeared as well while the cursor stayed visible. The next trial started 400 ms later: a new yellow square appeared and participants were asked to come back to this starting position. The lateral position of the yellow square varied across the workspace conditions. In the *ipsilateral workspace* condition, the yellow square appeared at a distance of 5 cm laterally from the median of the mirror in the direction of the opposite workspace. In the *contralateral workspace* condition, the yellow square appeared at a distance of 27.5 cm laterally from the median of the mirror in the same workspace. The different positions of the yellow square as starting position were made to keep the kinematics and the direction of the movement constant (see [34]). As a consequence, in the *ipsilateral workspace* condition, the yellow square appeared at a distance of 5 cm from the most medial lines (i.e. 0 cm), 16.25 cm from the intermediate lines (i.e. ±11.25 cm) and 27.5 cm from the most lateral lines (i.e. ±22.5 cm). Conversely, in the *contralateral workspace* condition, the yellow square appeared at a distance of 5 cm from the most lateral lines, 16.25 cm from the intermediate lines and 27.5 cm from the most medial lines. Therefore, for the analyses, the distance of the lines was coded relatively to the yellow square as *short*, *intermediate* and *long distance line* (*distance of the lines from the starting position*; Fig 2). It is noteworthy that when the static hand was positioned *inside the workspace*, it was placed below the line presented at the long distance from the starting yellow square (i.e. below the lateral line in the *ipsilateral workspace* condition, and the median line in the *contralateral workspace* condition). Therefore, participants saw their static hand below the long line distance when the shutters were open (*visible condition*). The task took approximatively 45 minutes.

Before and after the line bisection task, participants were asked to evaluate their pain by mean of a numerical rating scale (NRS) and their hand temperature was recorded with an infrared thermometer (Tempett, SENSELab, Sweden).

### Measures

The bisection bias was calculated by subtracting the position of the true midpoint of the line from the position where the line was actually crossed by the cursor, and expressed as percentage of the total length of each line. Since the different line lengths were used to diversify the task and avoid behavioral strategies, and since this variable was of no primary interest regarding the present hypotheses, the values were merged and averaged together for each of the 24 conditions (2 *workspaces* x 2 *visions of the hands* x 2 *positions of the static hand x 3 distances of the line from the starting position*).

Data were double coded for the analyses. In the first set of analyses, data from bisection measures were coded according to the left vs. right side of space relative to the participants, independently of the side of the CRPS’ affected hand, and independently of which hand was used to perform the task. A positive value indicated a rightward deviation in the bisection, and a negative value market a leftward deviation. In the second set of analyses, data were recoded according to the spatial direction of the bisection deviation regarding the pathological side of patients’ body, i.e. according to the side that was ipsilateral vs. contralateral to the affected/static hand. Indeed, some studies pointed out that cognitive biases in CRPS patients might be spatially locked to the side of space corresponding to the pathological limb position [17–19]. Similarly, for control participants, data were recoded according to the ipsilateral vs. contralateral side of their static hand. To this aim, deviations, measured in the group of participants whose right hand was static, were multiplied by -1 in order to flip and match their performances with those obtained in the group of participants whose static hand was left. Therefore, in this latter set of values, a positive value indicated a contralateral deviation (i.e. towards the unaffected limb) in the bisection, and a negative value marked an ipsilateral deviation (i.e. towards the affected limb).

In addition, all patients were also evaluated before the experiment with other clinical measures. The volume of both hands was measured using a hand volumeter (FEI 12–3504 Baseline Volumetric Measuring Device, Forearm Set, 6x6x24 Inch Cavity). Participants were instructed to keep their hand in water for 15 seconds (up the radiocarpal joint), three times in a row. The water displacement was measured in ml and then, the mean of the three measures was calculated for each hand. The temperature of both hands was also measured in degree Celsius on the hands’ dorsa (at the location of the first dorsal interosseous muscle) with an infrared thermometer (Tempett, SENSELab, Sweden). We calculated the absolute difference between the affected and the unaffected hand for hand volume as well as for skin temperature. The Disabilities of the Arm, Shoulder and Hand (DASH) questionnaire (Institute for Work & Health, Canada) was used to evaluate the physical symptoms of the CRPS patients as well as their abilities to perform daily-life activities (final score computed on 30 items). Patients were also asked to report the average pain felt over the last 2 days with a numeric rating scale ranging from 0–10 (with 0 = no pain and 10 = worst pain imaginable). Finally, 7 participants wore accelerometer watches (ActiGraph ASP-BTLE 2GB Activity Monitor, ActiGraph, United States) during three days allowing us to have a measure of the degree of immobilization and physical activity. We then calculated the average vector magnitude count for the affected side minus the count for the unaffected side, representing the difference in the amount of motor activity, as the vector magnitude is a measure of acceleration of all three axes. A negative score showed that the unaffected hand was moved more often than the affected hand, while a positive score indicated the reverse. The more values are far from 0, the greater is the activity imbalance between the two limbs. All these measures are reported in Table 2.

**Table 2:** Clinical characteristics of the CRPS participants. # = patient number; Pain = average pain over the last 2 days rated on a visually presented numeric rating scale ranging from 0 to 10; T° affected (C°) = temperature of the affected hand in degree Celsius; T° unaffected (C°) = temperature of the unaffected hand in degree Celsius; T° diff = temperature difference between the affected–unaffected hand in degree Celsius; Volume affected (ml) = volume of the affected hand in milliliter; Volume unaffected (ml) = volume of the unaffected hand in milliliter; Volume diff = volume difference between the affected–unaffected hand in milliliter; DASH score = score calculated from the Disabilities of the Arm, Shoulder and Hand (DASH) questionnaire; Activity affected-unaffected = Average vector magnitude count of the affected hand minus the count of the unaffected hand, as assessed with accelerometers, *nr* = data not recorded. Paired sample t-tests between the affected and unaffected hands for the volume and temperature parameters were not significant (all t(14) ≤ |.704|, *p* ≥ .493).

#	Pain	T° affected (C°)	T° unaffected (C°)	T° diff (°C)	Volume affected (ml)	Volume unaffected (ml)	Volume diff (ml)	DASH score (/100)	Activity affected-unaffected
01	4	31.7	30.3	+1.4	164.7	185	-20.3	30.8	*nr*
02	3	24.5	26.5	-2	156.6	151.3	+5.3	54.2	*nr*
03	5	31.2	32.6	-1.4	328.3	345	-16.7	60.8	*nr*
04	5	33.2	33.5	-0.3	156	165	-9	47.5	*nr*
05	5	24.6	25.8	-1.2	121.6	126.6	-5	40.8	-858.4
06	2	29.2	29.4	-0.2	170	156.6	+13.4	15.8	-816
07	1	28.2	27.2	+1	318.3	278.3	+40	54.1	-201.7
08	4	32	33.2	-1.2	151.3	150	+1.3	52.5	-83.1
09	2	33.5	33	+0.5	191.6	146.6	+45	60.8	*nr*
10	7	29.4	30	-0.6	383.3	363.3	+20	63.3	*nr*
11	7	35.6	34.3	+1.3	216.6	225	-8.4	81.7	-702.5
12	4	28.3	31.2	-2.9	303.3	291.6	+11.7	39.1	-1359.6
13	8	34.4	34.5	-0.1	121.6	140	-18.4	69.2	*nr*
14	8	30.8	30.9	-0.1	186.6	173.3	+13.3	44.1	113.7
15	3	35.4	35.1	+0.3	270	286.6	-16.6	12.5	*nr*

### Data analysis

To characterize whether the bisection deviated significantly from the midpoint of lines, the bisection bias measures were compared to zero for each condition and each group of participants by mean of one-sample t-tests. The only group factor used in this analysis is the one dissociating CRPS from control participants, and the data from the sub-groups build according to which hand was actively used were merged at this level of the analyses.

The effects of the different variables on the bisection biases was tested through an analysis of variance (ANOVA) with *workspace* (ipsilateral vs. contralateral), *vision of the hands* (visible vs. invisible), *position of the static hand* (inside vs. outside the workspace) and *distance of the line from the starting position* (short vs. intermediate vs. long) as within-participant factors, and *which hand is used* (left vs. right) and *group* (CRPS vs. control) as between-participant factors. Analyses were performed on the two sets of measures respectively (i.e. coding according to left vs. right sides and coding according to ipsilateral vs. contralateral sides). Effect sizes were measured using Cohen’s d for t-tests and partial Eta squared for ANOVAs. Greenhouse-Geisser corrections and contrast analyses were performed when needed and the significance level was set at p = .05. Corrections for multiple comparisons were not applied [35].

## Results

**Fig 4:**
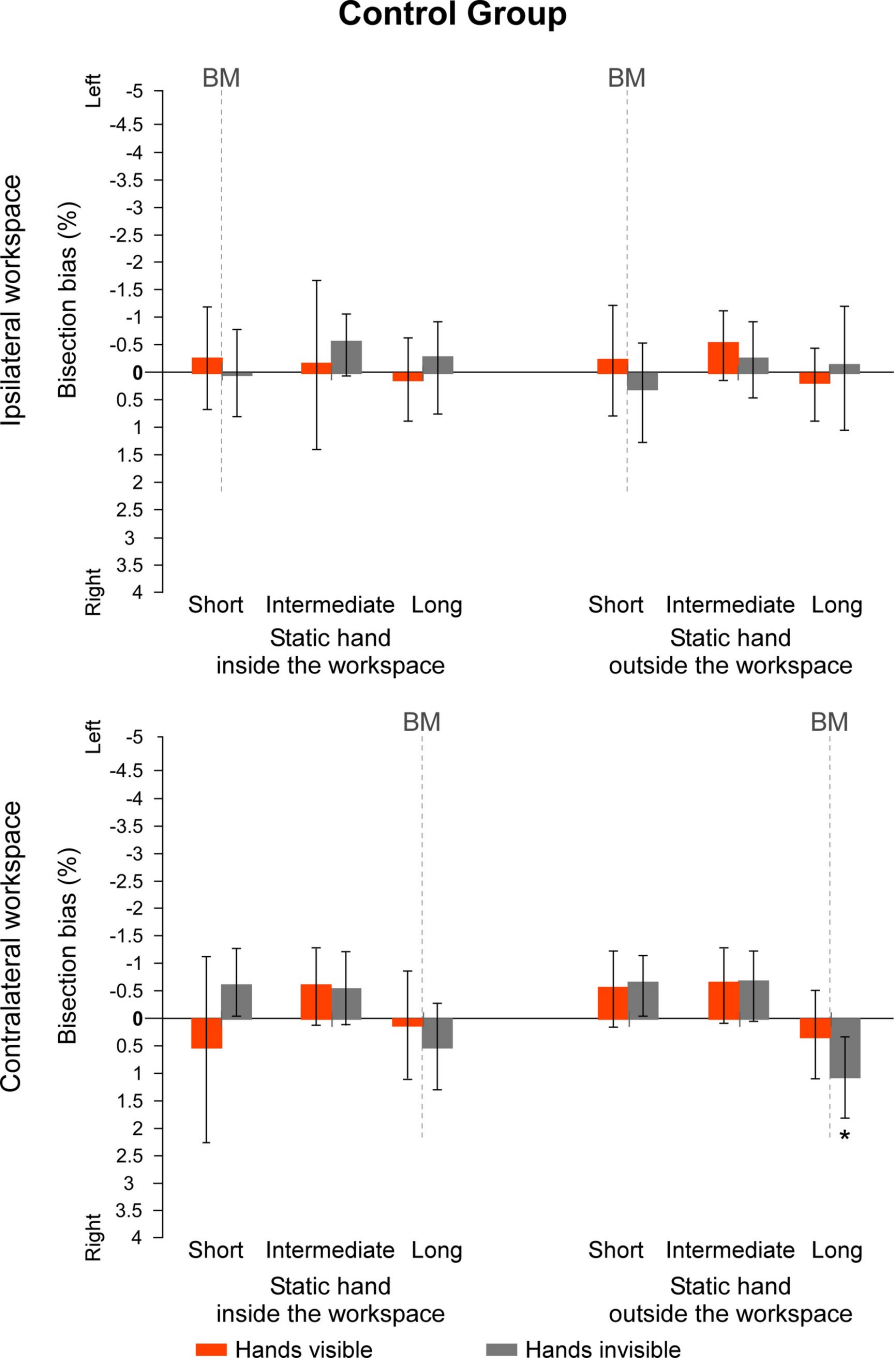
Mean bisection biases made by the control participants (spatial code). Values represent the mean differences between the estimation of the participants and the real midpoint of the lines, according to each of the *workspace*, *position of the static hand*, *vision of the hands* and *line distance* (short vs. intermediate vs. long) conditions. Direction of the bisection biases are coded relatively to the side of space (i.e. left- vs. rightward). Note that the line length factor is not represented in this figure. Dash lines at 0 cm correspond to the vertical median of the semi-reflexive screen to which participant’s body midline (BM) was aligned. The values of the two sub-groups defining which hand was used to perform the bisection were merged together. Values are expressed in terms of percentage of the total length of the lines. Negative values indicate leftward biases and positive values rightward biases. Error bars indicate the 95% confidence intervals adapted according to the method of Cousineau [36]. Asterisks illustrate the t-tests against zero that reach significance level (* .05 ≥ *p* > .01, ** *p* ≤ .01).

**Fig 5:**
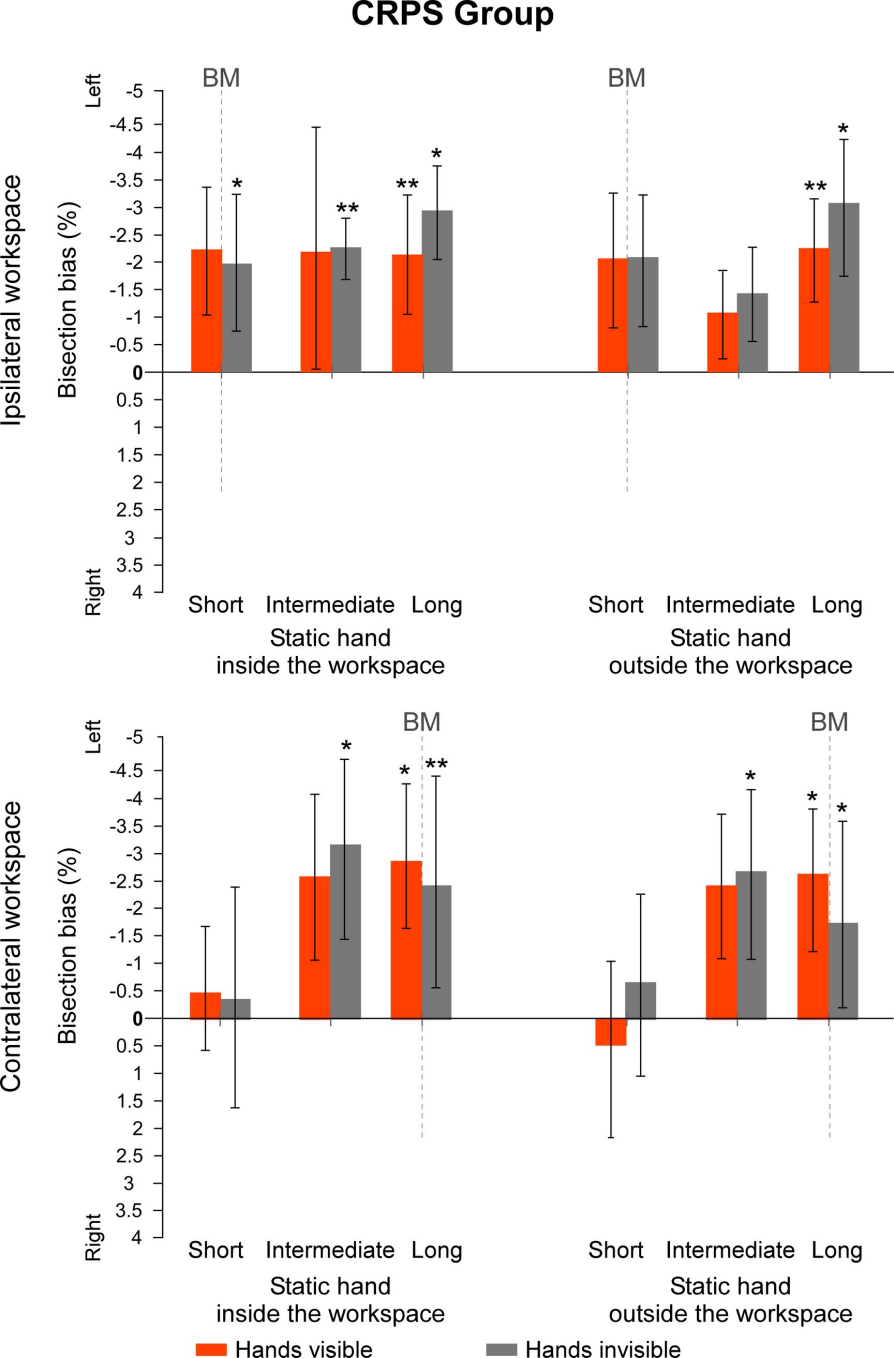
Mean bisection biases made by the CRPS participants (spatial code). Values represent the mean differences between the estimation of the participants and the real midpoint of the lines, according to each of the *workspace*, *position of the static hand*, *vision of the hands* and *line distance* (short vs. intermediate vs. long) conditions. Direction of the bisection biases are coded relatively to the side of space (i.e. left- vs. rightward). Note that the line length factor is not represented in this figure. Dash lines at 0 cm correspond to the vertical median of the semi-reflexive screen to which participant’s body midline (BM) was aligned. The values of the two sub-groups defining which hand was used to perform the bisection were merged together as this factor did not influence the direction of the biases in CRPS participants. Values are expressed in terms of percentage of the total length of the lines. Negative values indicate leftward biases and positive values rightward biases. Error bars indicate the 95% confidence intervals adapted according to the method of Cousineau [36]. Asterisks illustrate the t-tests against zero that reach significance level (* .05 ≥ *p* > .01, ** *p* ≤ .01).

Regarding the first set of values measuring the direction of the biases according to the left vs. right sides of space, data from the sub-groups *which hand is used* were merged together for each group. In the group of control participants, only one t-test revealed a bisection bias significantly different from 0 when lines were projected at a long distance in the contralateral workspace, with the hands invisible and the static hand outside the workspace (t(14) = 2.256, *p* = .041, d = .58). All the other values were not significantly different from 0 (all t(14) ≤ |1.541|, *p* ≥ .146, d ≤ .40) (Fig 4). By contrast, in the group of CRPS patients, all t-tests showed a significant deviation towards the left side of space when lines were projected at the longest distance from the starting position (all t(14) ≥ |2.280|, *p* ≤ .039, d ≥ .59). Additionally, t-tests on lines projected at an intermediate distance from the starting position were significantly different from 0 when hands were not visible (all t(14) ≥ |2.240|, *p* ≤ .042, d ≥ .58), except when performing the task with the static hand outside the ipsilateral workspace (t(14) = -1.543, p = .145, d = .40). None of the t-tests for lines at an intermediate distance in the visible condition were different from 0 (all t(14) ≤ |2.005|, *p* ≥ .065, d ≤ .52). Finally, lines projected at a short distance from the starting position were only significantly different from 0 when CRPS participants did the task with their hands invisible and their static hand inside the ipsilateral workspace (t(14) = -2.576, *p* = .022, d = .66). None of the other deviations for lines projected at a short distance from the starting position were significantly different from 0 (all t(14) ≤ |1.940|, *p* ≥ .073, d ≤ .50) (Fig 5). To summarize, while control participants did generally not show any significant bisection bias, CRPS participants showed leftward biases that reach significant level in half of the bisection conditions, especially when they had to move their unaffected hand over a long distance to reach the lines. Individual data can be found in supporting information (see S2 and S3 Figs).

**Fig 6:**
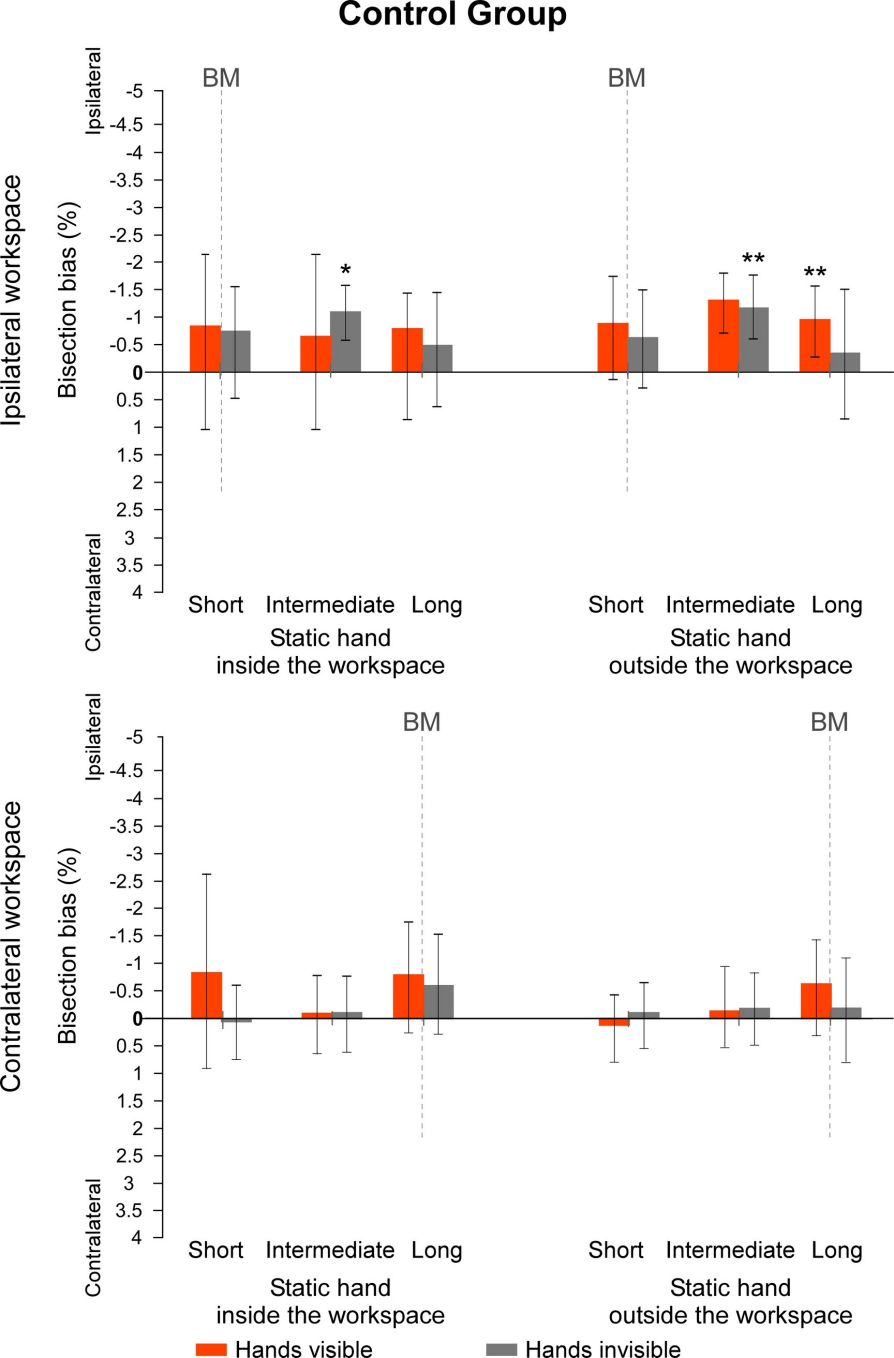
Mean bisection biases made by the control participants (hemibody code). Values represent the mean differences between the estimation of the participants and the real midpoint of the lines, according to each of the *workspace*, *position of the static hand*, *vision of the hands* and *line distance* (short vs. intermediate vs. long) conditions. Direction of the bisection biases are coded relatively to the static hand (i.e. ipsi- vs. contralateral). Note that the line length factor is not represented in this figure. Dash lines at 0 cm correspond to the vertical median of the semi-reflexive screen to which participant’s body midline (BM) was aligned. The values of the two sub-groups defining which hand was used to perform the bisection were merged together. Values are expressed in terms of percentage of the total length of the lines. Negative values indicate ipsilateral biases and positive values contralateral biases. Error bars indicate the 95% confidence intervals adapted according to the method of Cousineau [36]. Asterisks illustrate the t-tests against zero that reach significance level (* .05 ≥ *p* > .01, ** *p* ≤ .01).

**Fig 7:**
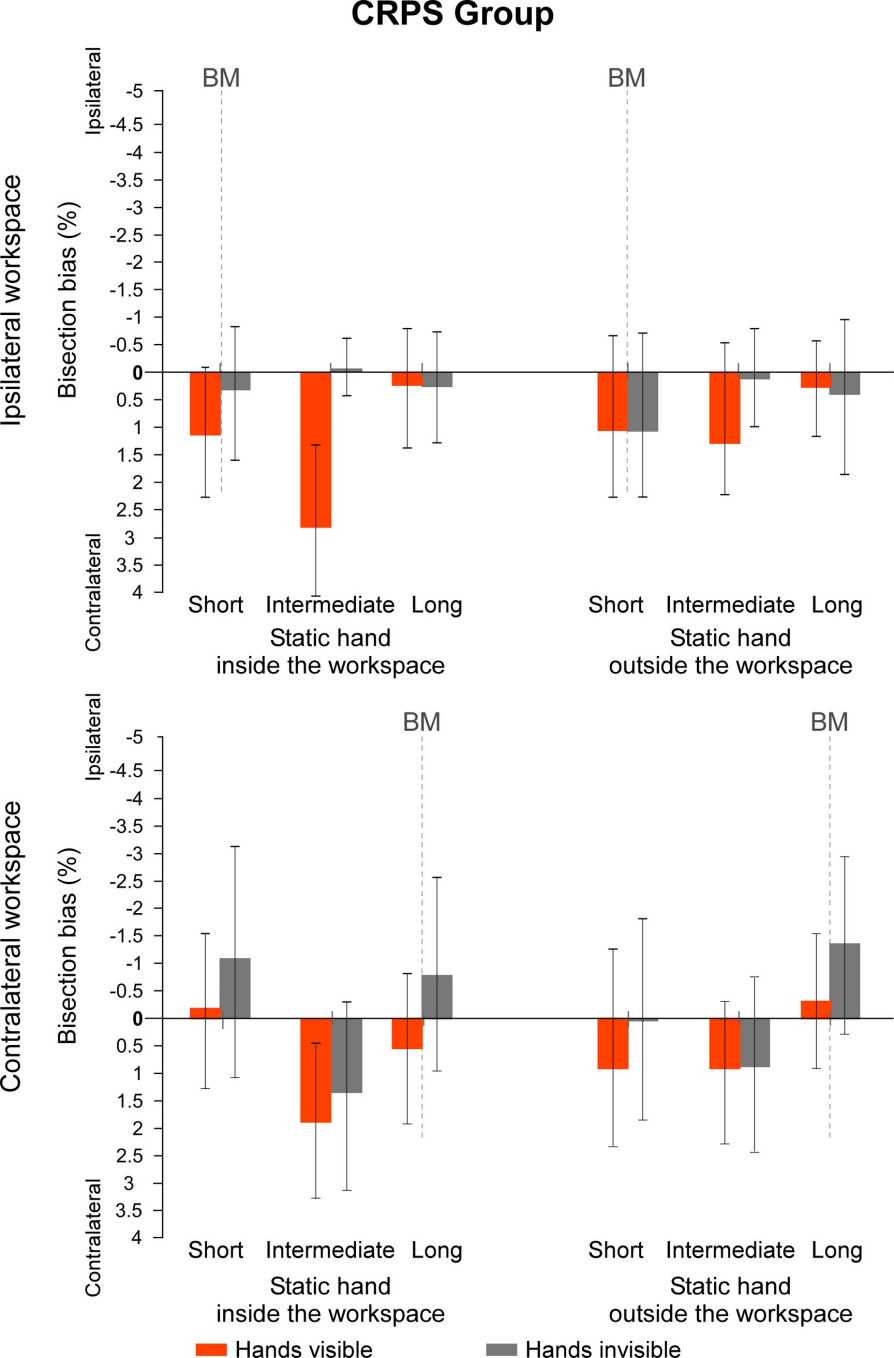
Mean bisection biases made by the CRPS participants (hemibody code). Values represent the mean differences between the estimation of the participants and the real midpoint of the lines, according to each of the *workspace*, *position of the static hand*, *vision of the hands* and *line distance* (short vs. intermediate vs. long) conditions. Direction of the bisection biases are coded relatively to the static hand, that is, the pathological hand (i.e. ipsi- vs. contralateral). Note that the line length factor is not represented in this figure. Dash lines at 0 cm correspond to the vertical median of the semi-reflexive screen to which participant’s body midline (BM) was aligned. The values of the two sub-groups defining which hand was used to perform the bisection were merged together as this factor did not influence the direction of the biases in CRPS participants. Values are expressed in terms of percentage of the total length of the lines. Negative values indicate ipsilateral biases and positive values contralateral biases. Error bars indicate the 95% confidence intervals adapted according to the method of Cousineau [36]. Asterisks illustrate the t-tests against zero that reach significance level (* .05 ≥ *p* > .01, ** *p* ≤ .01).

Regarding the second set of values measuring the direction of the biases according to the contralateral vs. ipsilateral sides of space to the static limb, data from the sub-groups *which hand is used* were again merged together for each group. In the control group, several t-test revealed a bisection bias significantly different from 0 when lines were projected at an intermediate distance in the ipsilateral workspace, with the hands invisible (all t(14) ≥ |2.435|, *p* ≤ .029, d ≥ .63) and, with the hands visible and the static hand outside the workspace (t(14) = |3.175,| *p* = .007, d = .82). For all other conditions, t-tests against 0 did not reach significance (all t(14) ≤ |2.077|, *p* ≥ .057, d ≤ .54) (Fig 6). By contrast, in the CRPS group, none of the t-tests made on the recoded data were significantly different from 0 (all t(14) ≤ |1.888|, *p* ≥ .080, d ≤ .49) (Fig 7). Taken together, analyses over the two sets of data suggest that CRPS participants tend to shift their bisection towards the left side of space, independently of which hand is affected.

The first ANOVA performed on the data coded according to the left vs. right sides of space is reported in Table 3 (only the comparisons including the *group* factor that reached significance level are shown; the full ANOVA is detailed in S1 Table). Analyses revealed a significant main effect of *group* (F(1, 26) = 5.483, *p* = .027, η^2^_p_ = .174), a factor that significantly interacted with several within-factors of the tasks, such as with *workspace* and the *distance of the line from the starting position* (F(1, 26) = 6.150, *p* = .004, η^2^_p_ = .191), *vision of the hand*s and the *which hand is used* (F(1, 26) = 8.606, *p* = .007, η^2^_p_ = .249) and, *workspace* and *which hand is used* (F(1, 26) = 4.320, *p* = .048, η^2^_p_ = .142).

**Table 3:** Significant results of the ANOVA involving the group factor with *workspace* (ipsilateral vs. contralateral), *vision of the hands* (visible vs. invisible), *position of the static hand* (inside vs. outside the workspace) and *distance of the line from the starting position* (short vs. intermediate vs. long) as within-participant factors, and *group* (CRPS vs. control) and *which hand is used* (left vs. right) as between-participant factors.

Factors	F	df	*p*	η^2^_p_
*Group*	5.483	1, 26	.027	.174
*Group × Line distance*	3.680	2, 52	.032	.124
*Group × Line distance × Workspace*	6.150	2, 52	.004	.191
*Group × Vision of the hands × Which hand is used*	8.606	1, 26	.007	.249
*Group × Workspace × Which hand is used*	4.320	1, 26	.048	.142

**Fig 8:**
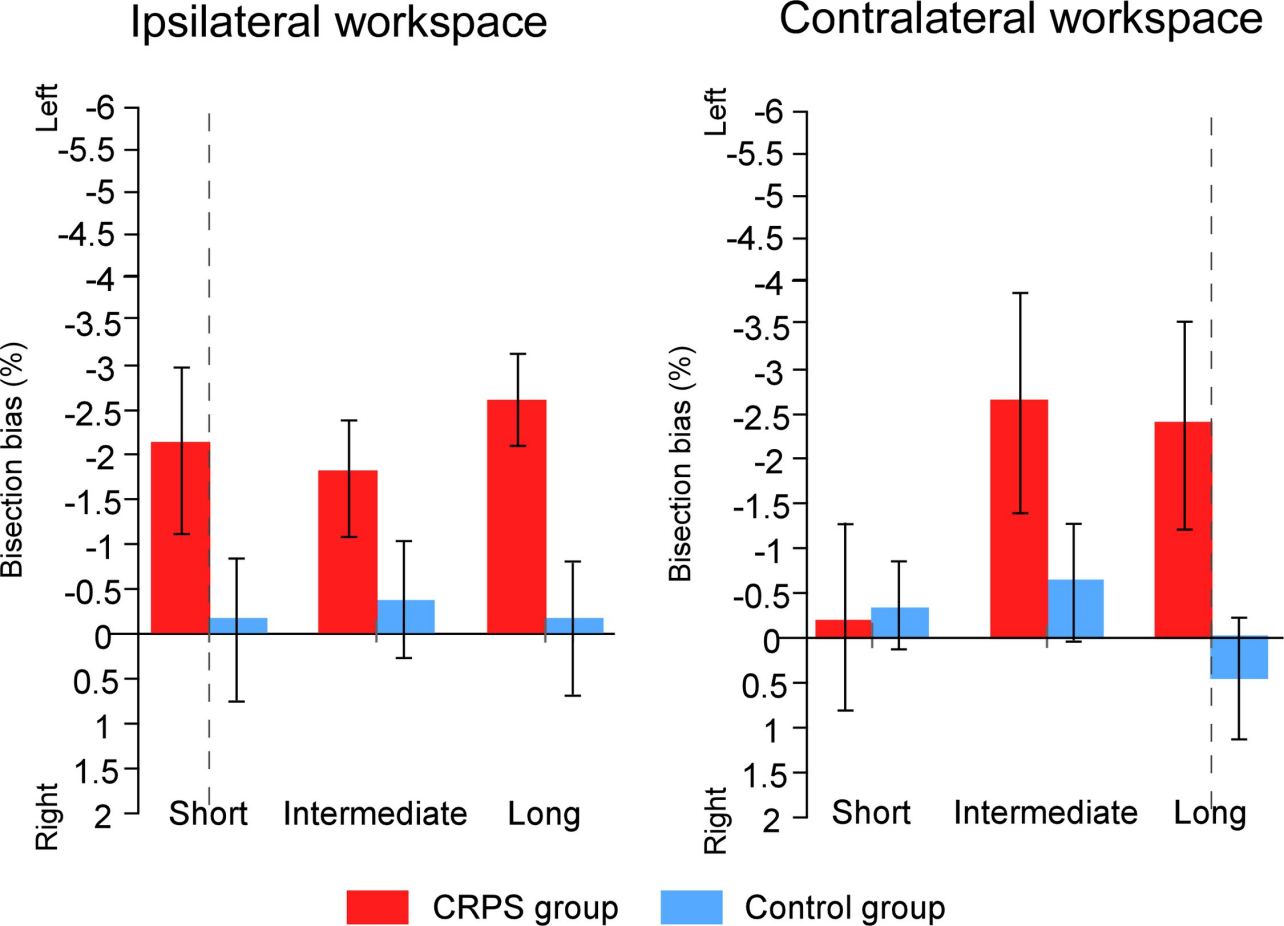
Mean bisection biases according to *line distance* and *workspace*. The graphs illustrate the comparison of the bisection biases between the CRPS participants (red bars) and the control participants (blue bars) according to the workspace into which the task was performed (contralateral vs. ipsilateral to the static hand) and the distance of the lines (short vs. intermediate vs. long), independently of the other variables. Direction of the bisection biases are coded relatively to the side of space (i.e. left- vs. rightward). Vertical dash lines correspond to the vertical median of the semi-reflexive screen to which participant’s body midline was aligned. Error bars represent the 95% confidence intervals adapted according to the method of Cousineau [36].

We first investigated the group difference from the ‘*group* x *distance of the line’* and ‘*group* × *distance of the line* × *workspace’* interactions according to the line distance, that is, the distance that the active hand had to cover to bisect a line. The group difference was significant for the long line distance (F(1, 26) = 10.772, *p* = .003, η^2^_p_ = .293), while it only approached significance level for the intermediate line distance (F(1,26) = 3.882, *p* = .060, η^2^_p_ = .130). This group difference showed that performances of the CRPS participants were more deviated towards the left side of space than those of the control participants for the long line distance (CRPS = -2.50% ± 3.76%; control = -0.22% ± 2.14%) and the intermediate line distance (CRPS = -2.22% ± 4.45%; control = -0.50% ± 2.17%) (Fig 8). Additionally, despite the absence of a main group difference for the short line distance (F(1,26) = 1.725, *p* = .201, η^2^_p_ = .062), we then found an interaction between *group* and *workspace* (F(1,26) = 11.540, *p* = .002, η^2^_p_ = .307) revealing a significant difference between CRPS and control participants for the short line distance only in the ipsilateral workspace (F(1,26) = 5.594, *p* = .026, η^2^_p_ = .177; CRPS = -2.07% ± 3.92%; control = -0.04% ± 1.85%). This interaction between *group* and *workspace* was not found for intermediate and long line distances (all F ≤ .585, *p* ≥.451, η2_p_ ≤ .022).

**Fig 9:**
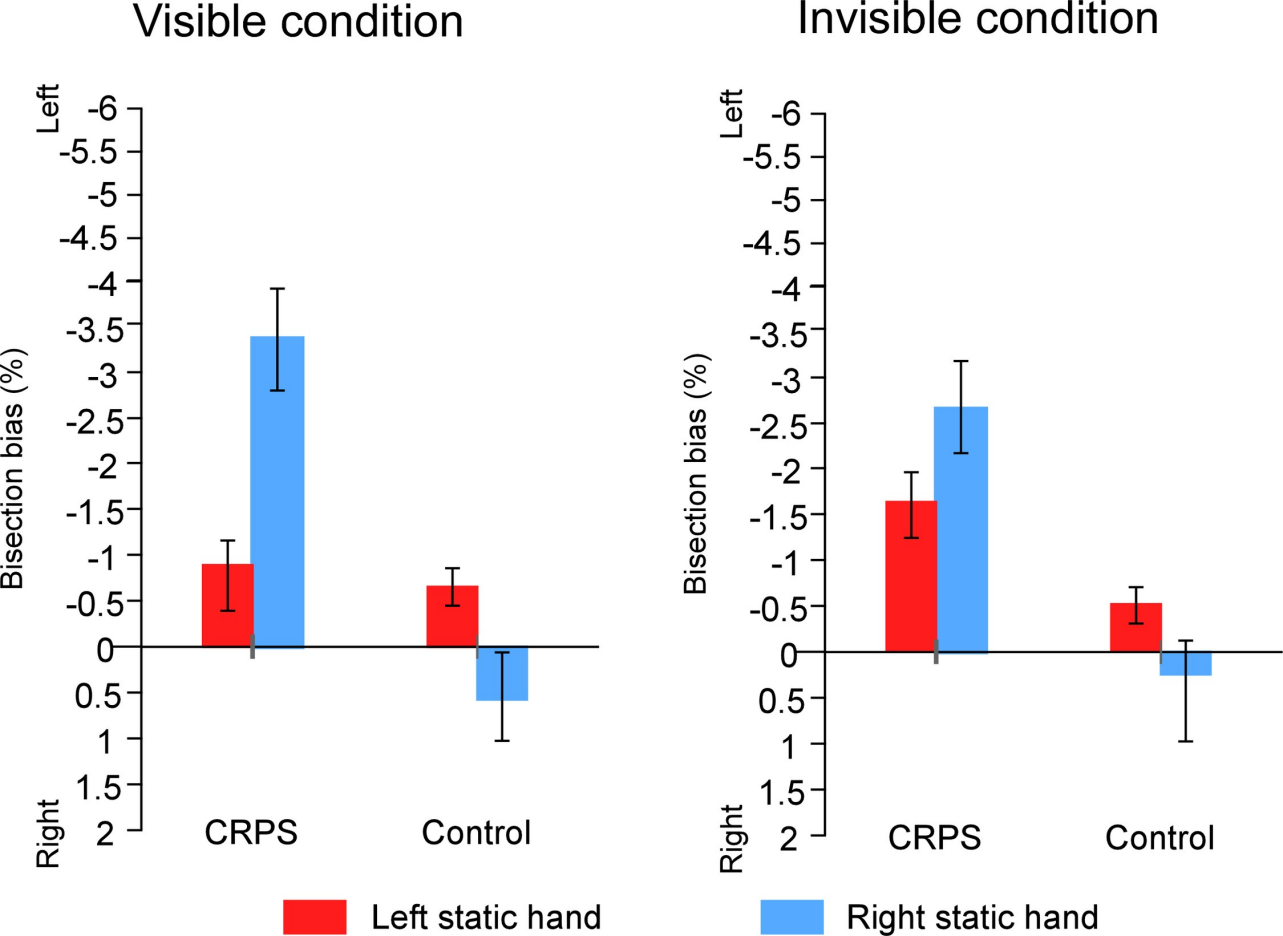
Mean bisection biases according to *vision of the hand* and *which hand is used*. The graphs illustrate the comparison of the bisection biases between the CRPS participants (left part of the graphs) and the control participants (right parts) according to whether the hands were visible or not and according to which of the hands was static (left hand: red bars; right hand: blue bars), independently of the other variables. Direction of the bisection biases are coded relatively to the side of space (i.e. left- vs. rightward). Error bars represent the 95% confidence intervals adapted according to the method of Cousineau [36].

We next investigated the difference between the two groups according to the visibility of the hands and the experimental setting (i.e. g*roup* x *vision of the hand* x *which hand is used*). CRPS participants’ bisection (M(visible) = -1.86% ± 4.22%; M(invisible) = -2.05% ± 3.76%) was significantly more deviated leftward as compared to control participants’ performance (M(visible) = -0.15% ± 2.28%; M(invisible) = -0.16% ± 2.02%), when the hands and setup were both visible (F(1,26) = 4.580, *p* = .042, η^2^_p_ = .150) and invisible (F(1,26) = 6.343, *p* = .018, η^2^_p_ = .196) (Fig 9). Nevertheless, the interaction between *group*, *vision* and *which hand is used* suggests that, in the visible condition, this difference between CRPS and control participants was not significant for the participants who performed the task with their right hand, i.e., the participants whose left hand remained static (F(1,16) = .053, *p* = .821, η2_p_ = .003).

**Fig 10:**
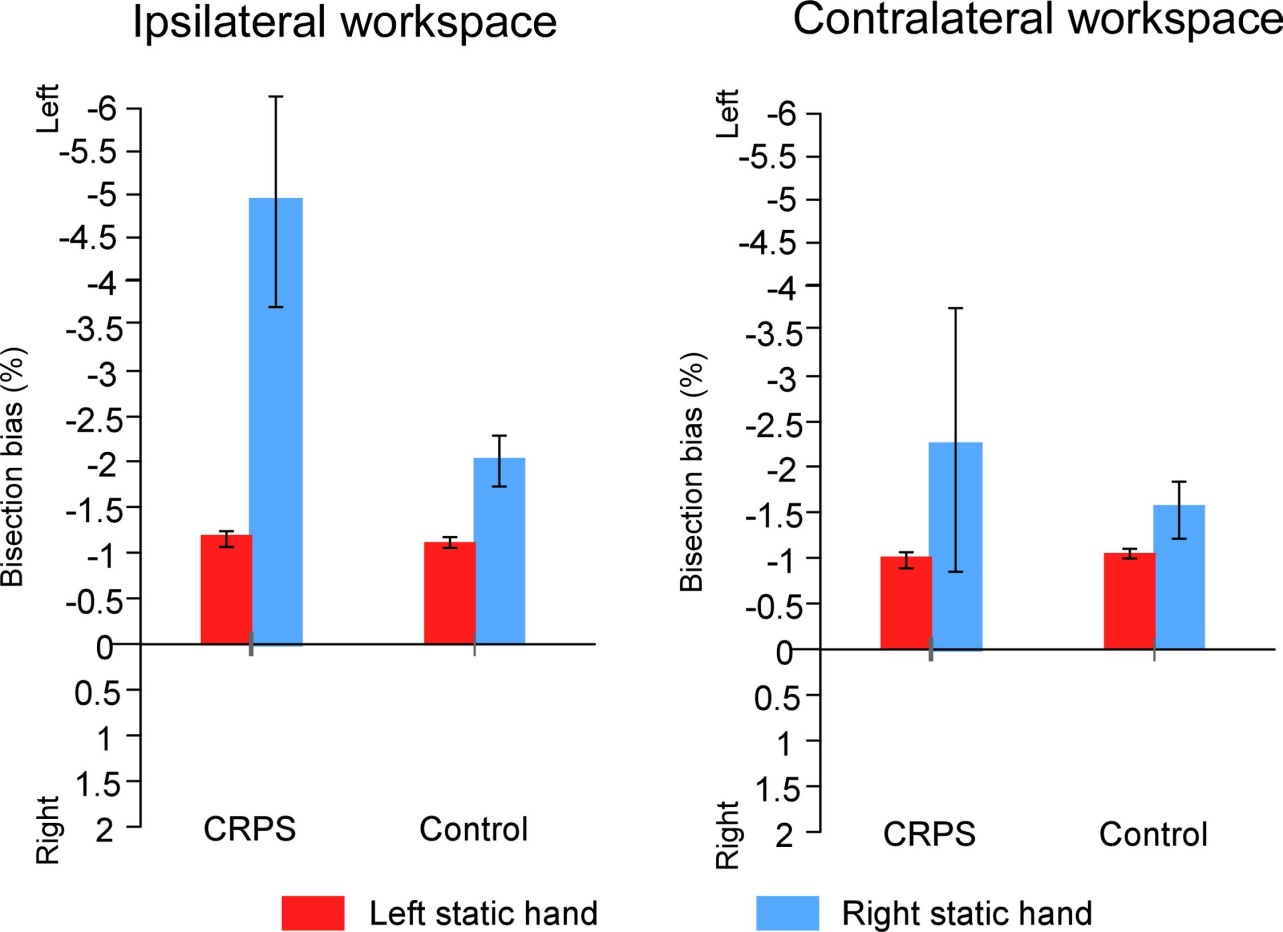
Mean bisection biases according to *workspace* and *which hand is used*. The graphs illustrate the comparison of the bisection biases between the CRPS participants (left part of the graphs) and the control participants (right parts) according to the workspace into which the task was performed (contralateral vs. ipsilateral to the static hand) and according to which of the hands was static (left hand: red bars; right hand: blue bars), independently of the other variables. Direction of the bisection biases are coded relatively to the side of space (i.e. left- vs. rightward). Error bars represent the 95% confidence intervals adapted according to the method of Cousineau [36].

Then, the group difference was analyzed regarding to the workspace in which the task was performed (i.e. *group* × *workspace* × w*hich hand is used*, Fig 10). CRPS participants (M(ipsilateral) = -2.13% ± 3.82%; M(contralateral) = -1.78% ± 4.16%) showed greater leftward bisection biases than control participants (M(ipsilateral) = -0.16% ± 2.12%; M(contralateral) = -0.15% ± 2.19%). However, such difference was significant in the ipsilateral workspace (F(1,26) = 6.491, *p* = .017, η^2^_p_ = .200) while it only tended to reach significance in the contralateral workspace (F(1,26) = 3.746, *p* = .064, η^2^_p_ = .126). Additionally, in the ipsilateral workspace, this difference was also modulated by the *which hand is used* factor (F(1,26) = 4.553, *p* = .042, η^2^_p_ = .149) suggesting that the group difference was nearly significant only when comparing participants having used the left hand to perform the task (F(1,10) = 4.623, *p* = .057, η^2^_p_ = .316; CRPS patients: M = -3.59% ± 4.83%; control participants: M = -0.76% ± 2.23%). This difference was not significant for participants having used their right hand (F(1,16) = .275, *p* = .607, η^2^_p_ = .017).

As a second step, the ANOVA was performed on the two groups separately. None of the aforementioned interactions were significant in the control group (all F ≤ 2.354, *p* ≥ .115, η^2^_p_ ≤ .153), suggesting that our results are mostly driven by CRPS participants’ data. Indeed, the ANOVA performed solely on CRPS patients showed an interaction between the *distance of the line* and the *workspace* (F(2,26) = 7.865, *p* = .002, η^2^_p_ = .377), indicating that the bisection bias was dependent of the distance between the line and the starting position, but only when the task was performed in the workspace contralateral to the static/affected hand. Indeed, the line distance effect was significant in the contralateral workspace condition (F(2,26) = 6.324, *p* = .006, η^2^_p_ = .327) but not in the ipsilateral workspace (F(2,26) = 1.263, *p* = .300, η^2^_p_ = .089). As such, this line distance effect showed that, in the contralateral workspace, CRPS participants’ bisection biases were smaller when the distance of the line from the starting position was short (M = -0.24% ± 2.89%) than intermediate (M = -2.7% ± 4.67%; F(1,13) = 10.593 , *p* = .006, η^2^_p_ = .449) and long (M = -2.4% ± 3.06%; F(1,13) = 7.782, *p* = .015, η^2^_p_ = .374). Bisection difference between the intermediate and long distances was not significant (F(1,13) = .602, *p* = .452, η^2^_p_ = .044). Similarly, when bisecting lines at a short distance, the bias was smaller when lines were projected in the workspace contralateral to the static/CRPS hand, than when projected in the ipsilateral workspace (contralateral = -0.24% ± 3.55%; ipsilateral = -2.07% ± 3.92%; F(1,13) = 14.706, *p* = .002, η^2^_p_ = .531).

The ANOVA on CRPS participants also showed a significant interaction between the *vision of the hands* and *which hand is used* factors (F(1,13) = 9.226, *p* = .010, η^2^_p_ = .415 ). Contrasts revealed that CRPS patients who used their right hand, i.e. patients with CRPS affecting their left hand, showed smaller biases when their hands were visible (M = -0.83% ± 2.38%) than when they were invisible (M = -1.62% ± 1.65%) (F(1,8) = 6.822, *p* = .031, η^2^_p_ = .460). Such difference was not significant in patients with CRPS affecting the right hand and so, using their left hand (F(1,5) = 3.114, *p* = .138, η^2^_p_ = .384).

The second ANOVA performed on the data coded according to the ipsilateral vs. contralateral sides of the static hand can be found in Table 4 (only the comparisons including the *group* factor that reached significance level are shown; the full ANOVA is detailed in S2 Table). Analyses revealed several interactions between the *group* factor and several other factors, such as with *Workspace*, *Line distance* and *Which hand is used* (F(2,52) = 6.150, *p* = .044, η^2^_p_ = .113), and the *vision of the hand*s (F(1, 26) = 8.606, *p* = .007, η^2^_p_ = .249).

**Table 4:** Significant results of the ANOVA involving the group factor with *workspace* (ipsilateral vs. contralateral), *vision of the hands* (visible vs. invisible), *position of the static hand* (inside vs. outside the workspace) and *distance of the line from the starting position* (short vs. intermediate vs. long) as within-participant factors, and *group* (CRPS vs. control) and *which hand is used* (left vs. right) as between-participant factors.

Factors	F	df	*p*	η^2^_p_
*Group × Which hand is used*	5.483	1, 26	.027	.174
*Group × Workspace*	4.320	1, 26	.048	.142
*Group × Vision of the hands*	8.606	1, 26	.007	.249
*Group × Line distance × Which hand is used*	3.680	2, 52	.032	.124
*Group × Workspace × Line distance × Which hand is used*	6.150	2, 52	.044	.113

**Fig 11:**
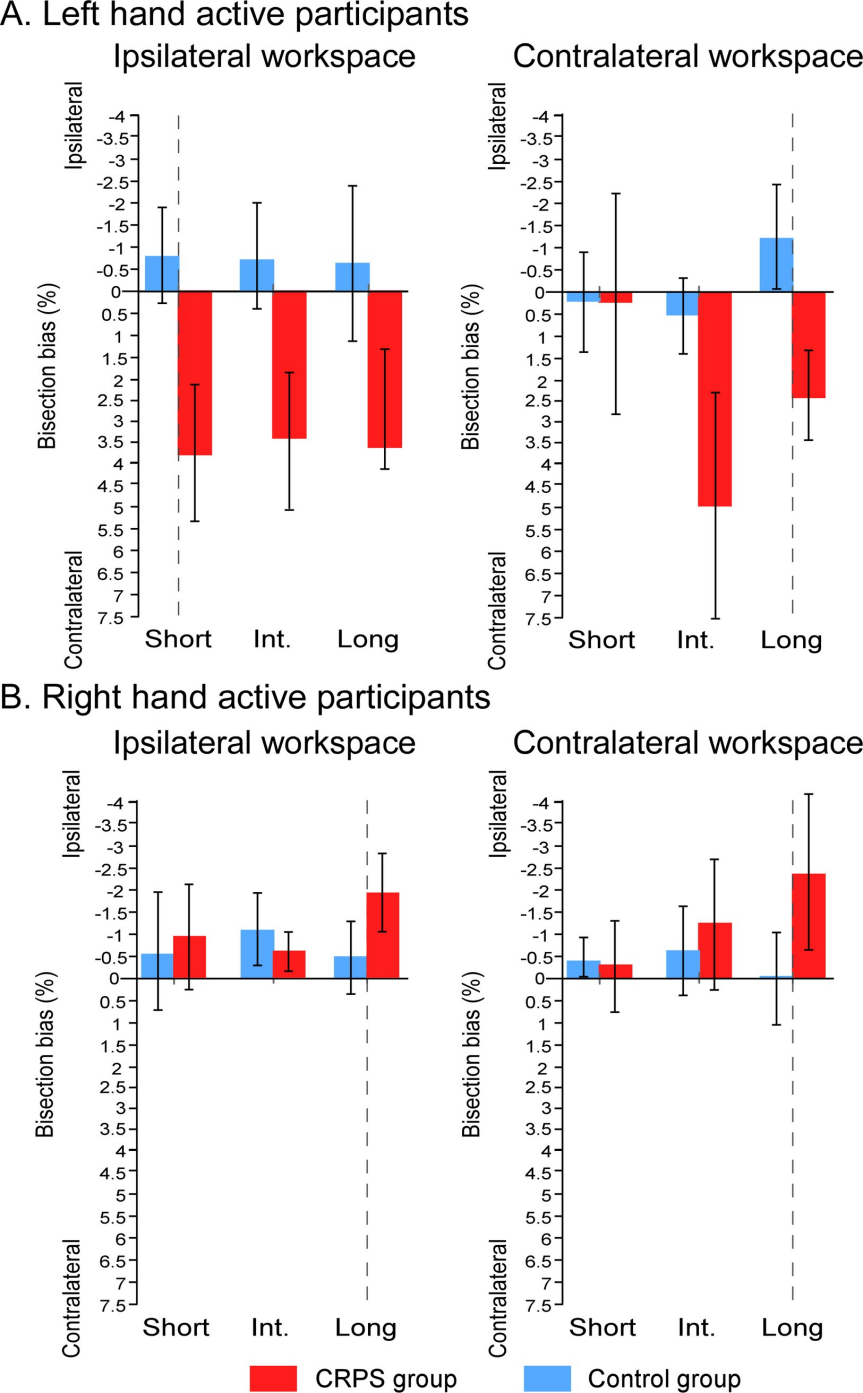
Mean bisection biases according to *workspace*, *line distance* and *which hand is used*. The graphs illustrate the comparison of the bisection biases between the CRPS participants (red bars) and the control participants (blue bars) according to the workspace into which the task was performed (contralateral vs. ipsilateral to the static hand) and the distance of the lines (short vs. intermediate vs. long) and which is used (A: left hand; B: right hand), independently of the other variables. Direction of the bisection biases are coded relatively to the static hand, that is, the pathological hand in CRPS participants (i.e. ipsi- vs. contralateral). Vertical dash lines correspond to the vertical median of the semi-reflexive screen to which participant’s body midline was aligned. Error bars represent the 95% confidence intervals adapted according to the method of Cousineau [36].

We first investigated the difference between the groups according to the experimental factors (i.e. *group × workspace × line distance × which hand is used* interaction, illustrated in Fig 11). It is worth noting that the interaction between the two group factors suggest that the bisection biases were made in opposite directions in the two sub-groups of CRPS patients: relatively to their respective controls, bisections of CRPS patients who used their left hand were deviated towards the side of space contralateral to their pathological right hand, while bisections of CRPS patients who used their right hand were deviated towards the side ipsilateral to their pathological left hand. In other words, the two sub-groups of patients showed significantly larger leftward bisection biases than their respective controls, as already highlighted in the previous sections. Accordingly, for lines projected at a short distance in the ipsilateral workspace, CRPS patients who used their left hand (M = 3.72% ± 3.36%) showed greater biases towards the contralateral side than their matched control participants (M = -0.82% ± 1.03%) (F(1,10) = 5.596, *p* = .040, η^2^_p_ = .359). CRPS patients who used their right hand (M = -2.39% ± 2.09%) made greater bisection biases towards the ipsilateral side than their matched control participants (M = -0.13% ± 1.34%), however, this time it was towards the ipsilateral side for lines projected at a long distance in the contralateral workspace (F(1,16) = 5.014, *p* = .040, η^2^_p_ = .239). Similarly, the direction difference is also illustrated by the fact that the bisection biases were significantly different between the two sub-groups of CRPS patients for most of the lines (F ≥ 5.596, *p* ≤ .040, η^2^_p_ ≥ .359) except for the lines at short distance of the ipsilateral workspace (F(1,13) = .085, *p* = .776, η^2^_p_ = .006).

This interaction also revealed that the previous results having shown smaller biases for the short distance lines projected in the contralateral workspace relatively to the other lines were mostly driven by the performances of the CRPS patients having used their left hand (i.e. right-hand CRPS) (*Workspace* x *Line Distance* interaction in this sub-group: F(2,10) = 6.147, *p* = .018, η^2^_p_ = .551; Line Distance effect in the contralateral workspace: F(1,5) = 6.522, *p* = .015, η^2^_p_ = .566; Line Distance effect in the ipsilateral workspace: F(1,5) = .072, *p* = .931, η^2^_p_ = .014; *Workspace* x *Line Distance* interaction in the right active hand CRPS sub-group: F(2,10) = 1.269, *p* = .308, η^2^_p_ = .137).

Finally, it was observed that the vision of the hands affected CRPS participants’ performances (F(1,13) = 9.226, *p* = .010, η^2^_p_ = .415), but not those of healthy volunteers (F(1,13) = .723, *p* = .411, η^2^_p_ = .053). CRPS patients’ biases were slightly but significantly larger when their hands were visible (M = 0.87% ± 2.63%) than when they were invisible (M = 0.10% ± 2.31%). This inversion of the effect with respect to the first ANOVA (i.e. larger biases in the invisible than the visible condition in left-hand CRPS participants) suggests that this result is not reliable due to the small number of participants in the subgroups.

## Discussion

The general aim of the present experiment was to investigate whether limited motor capacities observed in patients with CRPS affecting one upper-limb could be due to learned nonuse strategies (learned nonuse hypothesis, [20, 37]) or to motor difficulties similar to those described in HSN characterized by an inability to move their limbs toward objects in a particular direction [21, 22, 38–41]. To this aim, CRPS patients were asked to reach and point with their unaffected limb, holding a robotic handle, to the middle of lines projected horizontally in front of them, while their affected hand remained static. Their performance was compared to that of healthy volunteers matched individually to each CRPS participants. Overall, it was found that, when moving and reaching to targets with their unaffected limb, CRPS patients’ bisection was deviated towards the left side of the lines, for half of the line conditions, regardless of the different task manipulations, and regardless of which limb was affected. Indeed, when results were recoded according the side of the pathological hand (i.e. contra- vs. ipsilateral), these biases disappeared. In addition, CRPS patients’ bisections biases were significantly larger than those of control participants. Therefore, our data clearly do not support the learned nonuse hypothesis [11]. Indeed, if motor deficits observed in CRPS patients primarily resulted from learned strategies to avoid any movements with the affected limb, we should not have observed errors during reach-to-point movements as participants moved their unaffected hand to perform the task. As a consequence, we might conclude that CRPS patients probably present cognitive changes that could hamper their ability to move and direct their limbs to reach a target, including with the healthy limb. Similar motor difficulties affecting actions made with the healthy limb were previously shown during finger tapping [42], limb positioning [43], and drawing of geometrical figures [44] and circles [45]. However, it is worth noting that our experiment did not allow determining whether bisection biases resulted from either pure perceptual vs. motor deficits. Indeed, we cannot distinguish whether bisection errors are due to difficulties to pay attention and perceive the lines along their entire length or to adjust the correct movement to their midpoints (for a discussion, see [46, 47]).

Bisection biases at the line bisection task were already observed in previous CRPS studies. For example, Förderreuther et al. [16] showed that CRPS patients were biased at a paper-pencil version of the line bisection task when using their unaffected hand. However, this bisection deviation emerged only for patients with CRPS affecting the right hand and when the lines were presented in front of the patients or to their right side. In contrast to our results, in this case, bisection biases were rightward. On the contrary, Kolb et al. [15], Reid et al. [48] and Christophe et al. [29] did not evidence, in CRPS patients, any bias at the paper-pencil line bisection task whether the task was performed with the affected or the unaffected hand. However, Christophe et al. [28] also described the performance of a single case with CRPS affecting her left hand who showed leftward biases (i.e. towards the affected side of space), regardless of which hand was used to perform the task. However, when using her unaffected right hand, the patient presented a stronger leftward deviation to bisect lines to the right side of her body than when the task was performed in front or to the left side of her body. Finally, Reinersmann et al. [27] observed a group of CRPS participants who tended to slightly bias their bisection leftward, but their performance was not significantly different from bisection of healthy participants. During the same study, authors also evaluated the subjective representation of patient’s body midline. To test this, patients had to stop a moving dot when they estimated that it was crossing their midsagittal plane. When the task was performed in the dark, and so without any external cues, they found a systematic shift of subjective midline towards the left side in both patients with CRPS of the left hemibody and patients with CRPS of the right hemibody. Although these results contradict those of Sumitani et al. [12] and Sumitani et al. [19] who observed a displacement of subjective body midline towards the affected side and those of Christophe et al. [29] who showed no subjective body midline deficits, Reinersmann et al. [27]’ data are in agreement with the results of the present study. Indeed, when line bisections were significantly biased, it was systematically towards the left side of lines. Such deviation towards the left side of space has already been documented in neurologically healthy participants, including during line bisection tests ([49–52]; for a review see [53, 54]). This leftward spatial bias so-called pseudoneglect has been discussed as reflecting attentional asymmetry due to hemispheric dominance in spatial attention control [55] and an involvement of the right cortical hemisphere controlling the allocation of attention towards both the contralateral and ipsilateral hemispaces [56]. In line with this interpretation, the line bisection task has been shown to preferentially activate the right hemisphere [57, 58]. Such exacerbated pseudoneglect might contribute to the impairments affecting visuospatial attentional abilities of CRPS patients [27].

The second objective of our study aimed at testing whether bisection biases could depend on the spatial location of the lines in the workspace. Because the direction of cognitive deficits has been shown to be determined by the location of symptoms on the body, that is, by which hemibody is primarily affected by CRPS [17, 18, 59], we first hypothesized that bisection biases would be more important for the lines projected in the part of space corresponding to the affected hemibody (i.e. ipsilateral side) than for the lines projected in the side of space corresponding to the unaffected hemibody (i.e. contralateral side). A second hypothesis was based on the results from recent studies having shown that CRPS patients tend to bias the perception of visual stimuli to the disadvantage of those presented in the side of space corresponding to the affected part of the body, when the two visual stimuli are each presented close to one hand [17], and also that such bias can be influenced by the current position of the affected hand [18]. Based on these observations, we hypothesized that the largest bisection biases would be observed for lines projected in the close vicinity of the affected hand, that is, the lines projected just above the affected hand when that hand was placed inside the workspace (i.e. the long-distance lines). Regarding the first hypothesis, we did not observe any major effect of the position of the lines, i.e. whether the lines were presented in the side of space ipsilateral vs. contralateral to the affected part of the body. The only difference that we observed between the two workspaces is that the bisection of lines presented at a short distance in the contralateral workspace was significantly smaller than the bisection of other lines. But this latter result must be interpreted with caution. Indeed, for the other lines, the bisection was not different between the two workspaces, i.e. the deviation was always leftward. Moreover, the second set of analyses (i.e., contra- vs. ipsilateral coding) suggests that this effect is mostly present in one of the patient sub-groups, i.e. participants with CRPS affecting the right hand. In fact, the distance effect we observed in the contralateral workspace was already observed in healthy volunteers [26] and has been hypothesized to be related to different scaling of the movement as a consequence of the different curvatures of the movement between lines at short vs. longer distance [34].

The second hypothesis was addressed by manipulating the position of the to-be-bisected lines (by means of the *distance of the lines* conditions) and the position of the affected hand (i.e. inside vs. outside the workspace). As mention above, we hypothesized that we would observe larger bisection deviations when the affected hand was placed inside the workspace, especially for the lines projected on top of the affected hand, i.e. the lines at a long distance from the starting position when the hand was placed in the workspace. However, we did not observe a bisection difference depending on whether the affected hand was placed inside or outside the workspace, and the long-distance lines presented right above the pathological hand was not bisected differently than other lines. In other words, when moving in the workspace ipsilateral to the affected hand, bisection biases did not depend on whether the lines were presented close vs. farther away from the affected hand. Therefore, bisection biases of the CRPS patients do not seem to be related to the position of visual targets relatively to affected part of patients’ body and the actual position of the affected hand during the task.

Because bisection deviations seem to be less dependent on their position with regard to the patient’s body than expected, it could well be that CRPS patients’ cognitive symptoms can not only affect egocentric reference frames, but also allocentric reference frames. In egocentric reference frames, spatial positions are indeed coded with reference to the observer’s position, while in allocentric reference frames, an object’s position is spatially coded with regard to the location of other objects or to their own spatial structure, irrespective of the observer [31]. Studies in post-stoke neglect suggest that line bisection deviations reflect more often deficits affecting allocentric reference framing [60–62]. However, in most studies, unilateral cognitive deficits in CRPS have been interpreted as reflecting spatial cognition impairments related to the patients’ body (e.g. [12, 19, 27, 45, 48]). For instance, Reid et al. [48] recently suggested that observed representational, perceptual and motor deficits in CRPS might reflect a specific form of unilateral spatial cognition impairments affecting specifically somatic perception and the processing of bodily information in general. The present data contradicts this hypothesis, as we showed that spatial deviation was biased regardless of the position of the patients’ affected hand, suggesting that the bisection biases that we observed cannot be explained by impaired perception or representation of the body. Similarly, Filbrich et al. [17] showed that CRPS can specifically affect the perception of visual stimuli with no significant impact on the perception of tactile stimuli. Altogether, our findings suggest that cognitive difficulties in CRPS are likely to reflect more than specific somatospatial perceptual difficulties, and are thus probably more heterogeneous than originally described, such as in HSN after a stroke. For instance, authors highlighted that HSN can specifically affect body and somatosensory stimuli perception, while preserving the ability to perceive and react to external visual stimuli [63–68]. Somatospatial deficits are therefore not specific of CRPS. Other studies also reported dissociation in HSN between deficits affecting egocentric vs. allocentric reference frames [61, 69], and deficits affecting the ability to perceive visual stimuli either in the space close or far from the body [70, 71]. Similarly, studies on CRPS patients have described a variety of cognitive symptoms in this population [5, 13, 17, 18, 27–30, 43, 72]. Such variety in the symptomology could be the consequence of the various possibilities of adaptive strategies due to the heterogeneity of disease histories, patients’ cognitive profiles and other contextual factors. Indeed, these behavioral strategies that patients adopt to cope with pain can influence the cortical reorganization in the recovery process [1]. Altogether, these observations can lead us to reasonably think that we must also expect a greater variety of cognitive deficits in the CRPS population than originally expected.

In the present study, we observed that CRPS patients can display difficulties in bisecting lines with their unaffected hand regardless of the position of the lines relative to their body, thus discarding a learned non-use hypothesis. Further studies on cognitive deficits in CRPS will have to take into account the variability of expected cognitive deficits given the wide amount of spatial perceptual abilities and consider focusing on single-case studies.

## Supporting information

S1 Fig Flow diagram outlining participants. (PDF)

S2 Fig Individual bisection biases made by the control participants coded according to space (i.e. a left vs. right deviation). Values represent the recorded values differences between the estimation of the participants and the real midpoint of the lines, according to each of the *workspace (A = ipsilateral and B = contralateral)*, *position of the static hand*, *vision of the hands* and *line distance* (short vs. intermediate vs. long) conditions. Note that the line length factor is not represented in this figure. Dash lines at 0 cm correspond to the vertical median of the semi-reflexive screen to which participant’s body midline (BM) was aligned. Subjects plain lines correspond to participants whose static hand was left while subjects represented with dash lines were those with the right hand as static. Values are expressed in terms of percentage of the total length of the lines. Negative values indicate leftward biases and positive values rightward biases. Error bars indicate the 95% confidence intervals adapted according to the method of Cousineau [36]. Asterisks illustrate the t-tests against zero that reach significance level (* .05 ≥ *p* > .01, ** *p* ≤ .01).
(PDF)

S3 Fig Individual bisection biases made by the CRPS participants coded according to space (i.e. a left vs. right deviation). Values represent the recorded values differences between the estimation of the participants and the real midpoint of the lines, according to each of the *workspace (A = ipsilateral and B = contralateral)*, *position of the static hand*, *vision of the hands* and *line distance* (short vs. intermediate vs. long) conditions. Note that the line length factor is not represented in this figure. Dash lines at 0 cm correspond to the vertical median of the semi-reflexive screen to which participant’s body midline (BM) was aligned. Subjects plain lines correspond to participants whose static hand was left while subjects represented with dash lines were those with the right hand as static. Values are expressed in terms of percentage of the total length of the lines. Negative values indicate leftward biases and positive values rightward biases. Error bars indicate the 95% confidence intervals adapted according to the method of Cousineau [36]. Asterisks illustrate the t-tests against zero that reach significance level (* .05 ≥ *p* > .01, ** *p* ≤ .01).
(PDF)

S1 Table Results of the mixed ANOVA, coded according to the side of space (i.e. left vs. right), with workspace (ipsilateral vs. contralateral), vision of the hands (visible vs. invisible), position of the static hand (inside vs. outside the workspace) and distance of the line from the starting position (short vs. intermediate vs. long) as within-participant factors, and which hand is used (left vs. right) and group (CRPS vs. control) as between-participant factors.
(PDF)

S2 Table Results of the mixed ANOVA, coded according to the pathological side of patients’ body (i.e. ipsilateral vs. contralateral to the affected/static hand), with workspace (ipsilateral vs. contralateral), vision of the hands (visible vs. invisible), position of the static hand (inside vs. outside the workspace) and distance of the line from the starting position (short vs. intermediate vs. long) as within-participant factors, and which hand is used (left vs. right) and group (CRPS vs. control) as between-participant factors.
(PDF)
