# Supplementary figures and images for: Robot-assisted line bisection in patients with Complex Regional Pain Syndrome
Source: PLoS One. 2019 May 2;14(5):e0213732. doi: 10.1371/journal.pone.0213732 (PMC6497371; doi:10.1371/journal.pone.0213732)

## Supporting information

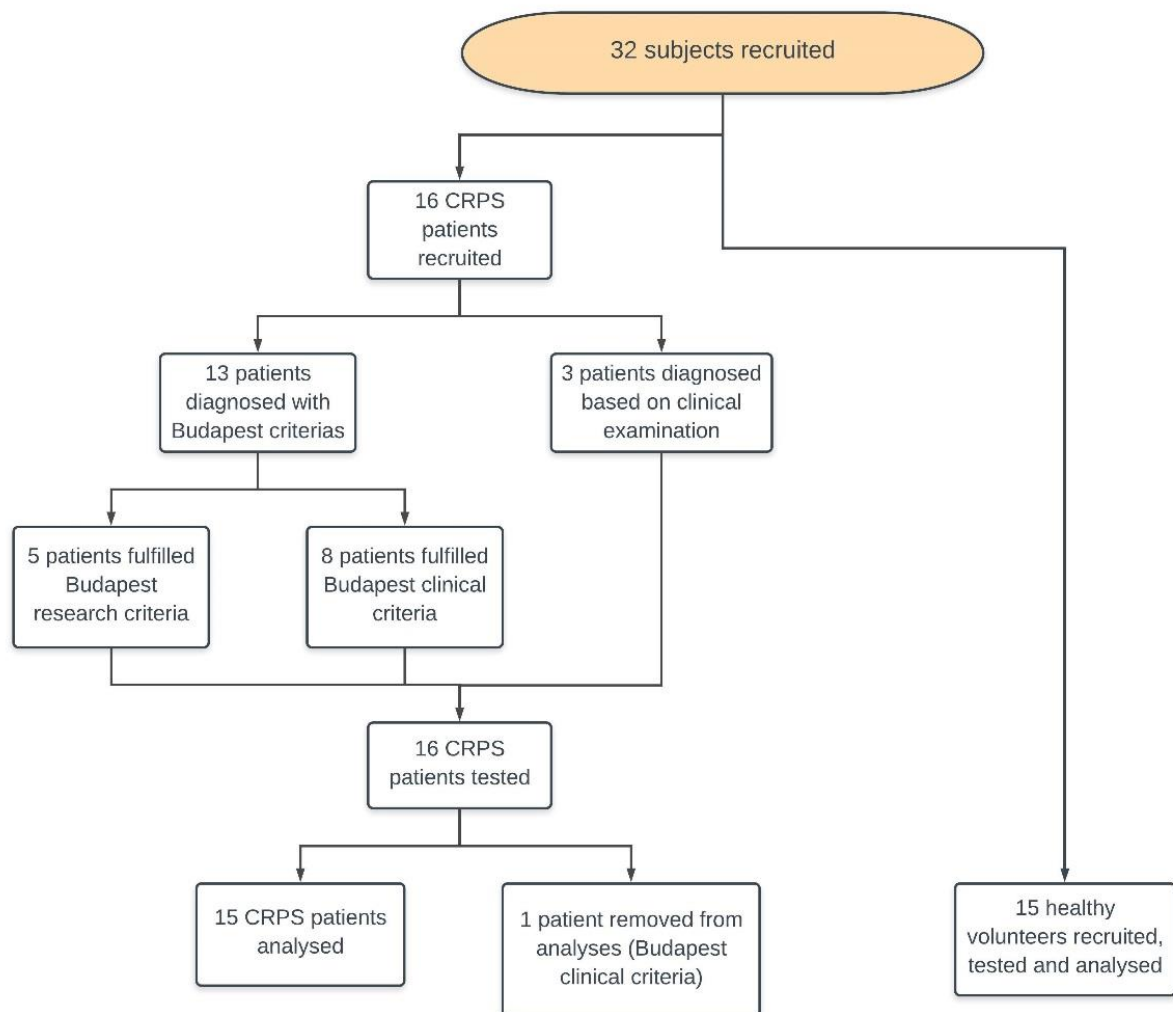

**S1 Fig.** Flow diagram outlining participants.

Supplement: S1 Fig — (PDF) [file pone.0213732.s001.pdf]
