# Supplementary material for: Robot-assisted line bisection in patients with Complex Regional Pain Syndrome
Source: PLoS One. 2019 May 2;14(5):e0213732. doi: 10.1371/journal.pone.0213732 (PMC6497371; doi:10.1371/journal.pone.0213732)
Supplement: S2 Fig — Values represent the recorded values differences between the estimation of the participants and the real midpoint of the lines, according to each of the workspace (A = ipsilateral and B = contralateral), position of the static hand, vision of the hands and line distance (short vs. intermediate vs. long) conditions. Note that the line length factor is not represented in this figure. Dash lines at 0 cm correspond to the vertical median of the semi-reflexive screen to which participant’s body midline (BM) was aligned. Subjects plain lines correspond to participants whose static hand was left while subjects represented with dash lines were those with the right hand as static. Values are expressed in terms of percentage of the total length of the lines. Negative values indicate leftward biases and positive values rightward biases. Error bars indicate the 95% confidence intervals adapted according to the method of Cousineau [36]. Asterisks illustrate the t-tests against zero that reach significance level (* .05 ≥ p > .01, ** p ≤ .01). (PDF) [file pone.0213732.s002.pdf]

Supporting information

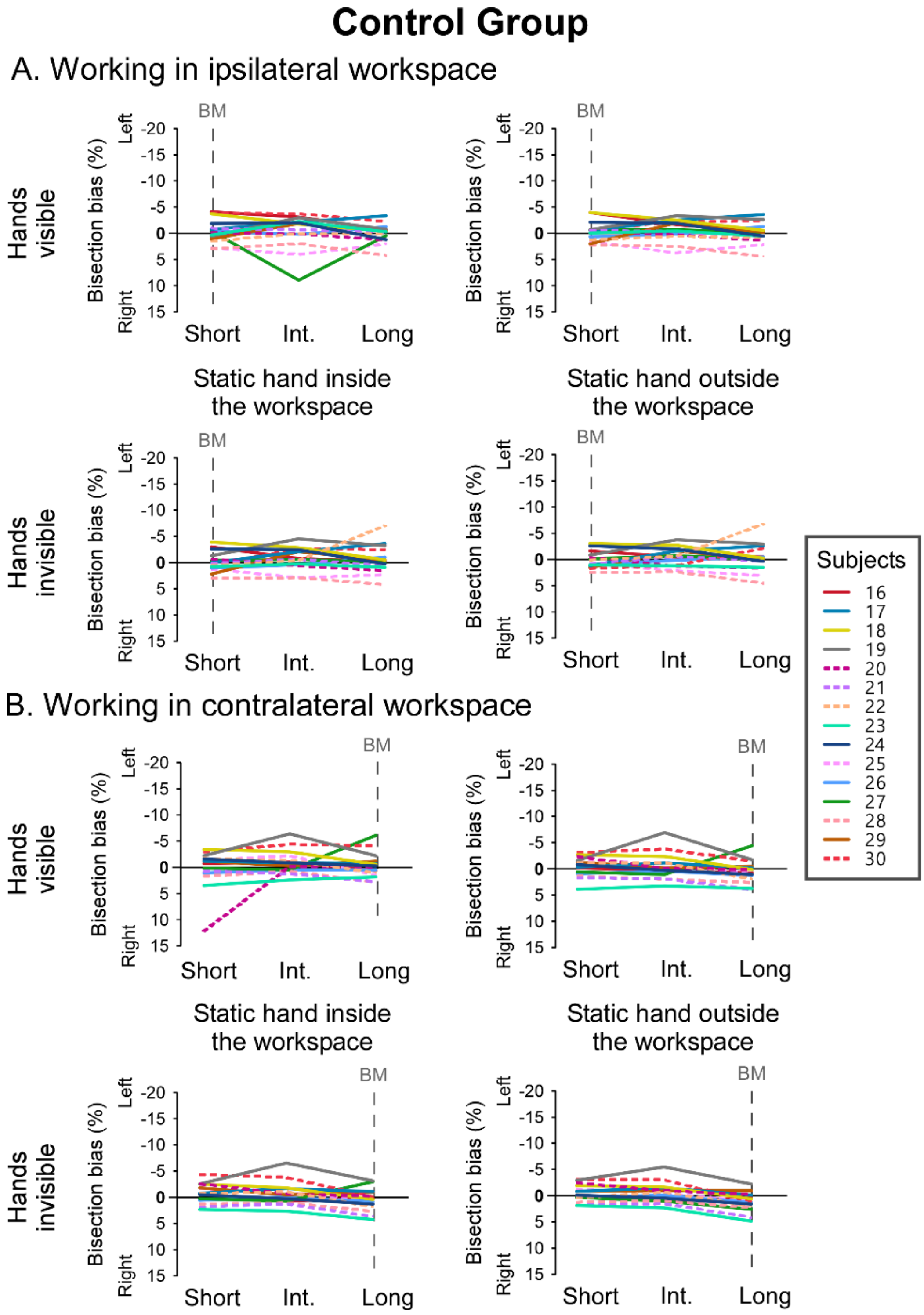

S2 Fig. Individual bisection biases made by the control participants coded according to space (i.e. a left vs. right deviation). Values represent the recorded values differences between

the estimation of the participants and the real midpoint of the lines, according to each of the *workspace (A = ipsilateral and B = contralateral), position of the static hand*, *vision of the hands* and *line distance* (short vs. intermediate vs. long) conditions. Note that the line length factor is not represented in this figure. Dash lines at 0 cm correspond to the vertical median of the semi-reflexive screen to which participant's body midline (BM) was aligned. Subjects plain lines correspond to participants whose static hand was left while subjects represented with dash lines were those with the right hand as static. Values are expressed in terms of percentage of the total length of the lines. Negative values indicate leftward biases and positive values rightward biases. Error bars indicate the 95% confidence intervals adapted according to the method of Cousineau [36]. Asterisks illustrate the t-tests against zero that reach significance level (* $.05 \geq p > .01$, ** $p \leq .01$).
