# Supplementary material for: Robot-assisted line bisection in patients with Complex Regional Pain Syndrome
Source: PLoS One. 2019 May 2;14(5):e0213732. doi: 10.1371/journal.pone.0213732 (PMC6497371; doi:10.1371/journal.pone.0213732)
Supplement: S1 Table — Results of the mixed ANOVA, coded according to the side of space (i.e. left vs. right), with workspace (ipsilateral vs. contralateral), vision of the hands (visible vs. invisible), position of the static hand (inside vs. outside the workspace) and distance of the line from the starting position (short vs. intermediate vs. long) as within-participant factors, and which hand is used (left vs. right) and group (CRPS vs. control) as between-participant factors. (PDF) [file pone.0213732.s004.pdf]

Supporting information

S1 Table. Results of the mixed ANOVA, coded according to the side of space (i.e. left vs. right), with *workspace* (ipsilateral vs. contralateral), *vision of the hands* (visible vs. invisible), *position of the static hand* (inside vs. outside the workspace) and *distance of the line from the starting position* (short vs. intermediate vs. long) as within-participant factors, and *which hand is used* (left vs. right) and *group* (CRPS vs. control) as between-participant factors.

Factors	F	*p*	η^2_p
Group	5.483	.027	.174
Which hand is used	0.205	.655	.008
Group × Which hand is used	2.559	.122	.090
Workspace	0.479	.495	.018
Workspace × Group	1.110	.302	.041
Workspace × Which hand is used	0.035	.853	.001
Workspace × Group × Which hand is used	4.320	.048	.142
Vision of the hands	0.041	.841	.002

Vision of the hands × Group	0.000	.989	.000
Vision of the hands × Which hand is used	3.632	.068	.123
Vision of the hands × Group × Which hand is used	8.606	.007	.249
Position of the static hand	3.773	.063	.127
Position of the static hand × Group	2.826	.105	.098
Position of the static hand × Which hand is used	0.437	.515	.017
Position of the static hand × Group × Which hand is used	0.840	.368	.031
Line distance	2.905	.064	.100
Line distance × Group	3.680	.032	.124
Line distance × Which hand is used	2.298	.111	.081
Line distance × Group × Which hand is used	1.948	.153	.070
Workspace × Vision of the hands	0.046	.832	.002
Workspace × Vision of the hands × Group	0.105	.749	.004
Workspace × Vision of the hands × Which hand is used	0.008	.931	.000
Workspace × Vision of the hands × Group ×× Which hand is used	0.013	.908	.001

Workspace × Position of the static hand	0.069	.795	.003
Workspace × Position of the static hand × Group	0.438	.514	.017
Workspace × Position of the static hand × Which hand is used	0.309	.583	.012
Workspace × Position of the static hand × Group × Which hand is used	0.996	.327	.037
Vision of the hands × Position of the static hand	0.082	.777	.003
Vision of the hands × Position of the static hand × Group	2.736	.110	.095
Vision of the hands × Position of the static hand × Which hand is used	1.712	.202	.062
Vision of the hands × Position of the static hand × Group ×Which hand is used	2.225	.148	.079
Workspace × Vision of the hands × Position of the static hand	0.377	.544	.014
Workspace × Vision of the hands × Position of the static hand × Group	0.007	.935	.000
Workspace × Vision of the hands × Position of the static hand × Which hand is used	3.625	.068	.122
Workspace × Vision of the hands × Position of the static hand × Group × Which hand is used	0.027	.871	.001
Workspace × Line distance	5.621	.006	.178
Workspace × Line distance × Group	6.150	.004	.191
Workspace × Line distance × Which hand is used	3.318	.044	.113

Workspace × Line distance × Group × Which hand is used	1.398	.256	.051
Vision of the hands × Line distance	0.085	.918	.003
Vision of the hands × Line distance × Group	0.022	.978	.001
Vision of the hands × Line distance × Which hand is used	0.920	.405	.034
Vision of the hands × Line distance × Group × Which hand is used	0.032	.968	.001
Workspace × Vision of the hands × Line distance	5.188	.020	.166
Workspace × Vision of the hands × Line distance × Group	0.474	.625	.018
Workspace × Vision of the hands × Line distance × Which hand is used	4.250	.020	.140
Workspace × Vision of the hands × Line distance × Group × Which hand is used	2.394	.101	.084
Position of the static hand × Line distance	1.740	.186	.063
Position of the static hand × Line distance × Group	0.887	.418	.033
Position of the static hand × Line distance × Which hand is used	6.787	.002	.207
Position of the static hand × Line distance × Group × Which hand is used	1.800	.175	.065
Workspace × Position of the static hand × Line distance	2.788	.071	.097
Workspace × Position of the static hand × Line distance × Group	1.966	.150	.070

Workspace × Position of the static hand × Line distance × Which hand is used	2.319	.108	.082
Workspace × Position of the static hand × Line distance × Group × Which hand is used	1.426	.249	.052
Vision of the hands × Position of the static hand × Line distance	0.068	.892	.003
Vision of the hands × Position of the static hand × Line distance × Group	1.101	.340	.041
Vision of the hands × Position of the static hand × Line distance × Which hand is used	0.711	.496	.027
Vision of the hands × Position of the static hand × Line distance × Group × Which hand is used	0.671	.515	.025
Workspace × Vision of the hands × Position of the static hand × Line distance	0.001	.999	.000
Workspace × Vision of the hands × Position of the static hand × Line distance × Group	1.951	.152	.070
Workspace × Vision of the hands × Position of the static hand × Line distance × Which hand is used	0.710	.497	.027
Workspace × Vision of the hands × Position of the static hand × Line distance × Group × Which hand is used	0.512	.602	.019
