# Supplementary material for: Robot-assisted line bisection in patients with Complex Regional Pain Syndrome
Source: PLoS One. 2019 May 2;14(5):e0213732. doi: 10.1371/journal.pone.0213732 (PMC6497371; doi:10.1371/journal.pone.0213732)
Supplement: S2 Table — Results of the mixed ANOVA, coded according to the pathological side of patients’ body (i.e. ipsilateral vs. contralateral to the affected/static hand), with workspace (ipsilateral vs. contralateral), vision of the hands (visible vs. invisible), position of the static hand (inside vs. outside the workspace) and distance of the line from the starting position (short vs. intermediate vs. long) as within-participant factors, and which hand is used (left vs. right) and group (CRPS vs. control) as between-participant factors. (PDF) [file pone.0213732.s005.pdf]

Supporting information

S2 Table. Results of the mixed ANOVA, coded according to the pathological side of patients' body (i.e. ipsilateral vs. contralateral to the affected/static hand), with *workspace* (ipsilateral vs. contralateral), *vision of the hands* (visible vs. invisible), *position of the static hand* (inside vs. outside the workspace) and *distance of the line from the starting position* (short vs. intermediate vs. long) as within-participant factors, and *which hand is used* (left vs. right) and *group* (CRPS vs. control) as between-participant factors.

Factors	F	*p*	η^2_p
Group	2.559	.122	.090
Which hand is used	6.063	.021	.189
Group × Which hand is used	5.483	.027	.174
Workspace	0.035	.853	.001
Workspace × Group	4.320	.048	.142
Workspace × Which hand is used	0.479	.495	.018
Workspace × Group × Which hand is used	1.110	.302	.041
Vision of the hands	3.632	.068	.123

Vision of the hands × Group	8.606	.007	.249
Vision of the hands × Which hand is used	0.041	.841	.002
Vision of the hands × Group × Which hand is used	0.000	.989	.000
Position of the static hand	0.437	.515	.017
Position of the static hand × Group	0.840	.368	.031
Position of the static hand × Which hand is used	3.773	.063	.127
Position of the static hand × Group × Which hand is used	2.826	.105	.098
Line distance	2.298	.111	.081
Line distance × Group	1.948	.153	.070
Line distance × Which hand is used	2.905	.064	.100
Line distance × Group × Which hand is used	3.680	.032	.124
Workspace × Vision of the hands	0.008	.931	.000
Workspace × Vision of the hands × Group	0.013	.908	.001
Workspace × Vision of the hands × Which hand is used	0.046	.832	.002
Workspace × Vision of the hands × Group × Which hand is used	0.103	.749	.004

Workspace × Position of the static hand	0.309	.583	.012
Workspace × Position of the static hand × Group	0.996	.327	.037
Workspace × Position of the static hand × Which hand is used	0.069	.795	.003
Workspace × Position of the static hand × Group × Which hand is used	0.438	.514	.017
Vision of the hands × Position of the static hand	1.712	.202	.062
Vision of the hands × Position of the static hand × Group	2.225	.148	.079
Vision of the hands × Position of the static hand × Which hand is used	0.082	.777	.003
Vision of the hands × Position of the static hand × Group ×Which hand is used	2.736	.110	.095
Workspace × Vision of the hands × Position of the static hand	3.625	.068	.122
Workspace × Vision of the hands × Position of the static hand × Group	0.027	.871	.001
Workspace × Vision of the hands × Position of the static hand × Which hand is used	0.377	.544	.014
Workspace × Vision of the hands × Position of the static hand × Group × Which hand is used	0.007	.935	.000
Workspace × Line distance	3.318	.044	.113
Workspace × Line distance × Group	1.398	.256	.051
Workspace × Line distance × Which hand is used	5.621	.006	.178

Workspace × Line distance × Group × Which hand is used	6.150	.004	.191
Vision of the hands × Line distance	0.920	.405	.034
Vision of the hands × Line distance × Group	0.032	.968	.001
Vision of the hands × Line distance × Which hand is used	0.085	.918	.003
Vision of the hands × Line distance × Group × Which hand is used	0.022	.978	.001
Workspace × Vision of the hands × Line distance	4.250	.036	.140
Workspace × Vision of the hands × Line distance × Group	2.394	.101	.084
Workspace × Vision of the hands × Line distance × Which hand is used	5.188	.020	.166
Workspace × Vision of the hands × Line distance × Group × Which hand is used	0.474	.625	.018
Position of the static hand × Line distance	6.787	.002	.207
Position of the static hand × Line distance × Group	1.800	.175	.065
Position of the static hand × Line distance × Which hand is used	1.740	.186	.063
Position of the static hand × Line distance × Group × Which hand is used	0.887	.418	.033
Workspace × Position of the static hand × Line distance	2.319	.108	.082
Workspace × Position of the static hand × Line distance × Group	1.426	.249	.052

Workspace × Position of the static hand × Line distance × Which hand is used	2.788	.071	.097
Workspace × Position of the static hand × Line distance × Group × Which hand is used	1.966	.150	.070
Vision of the hands × Position of the static hand × Line distance	0.711	.496	.027
Vision of the hands × Position of the static hand × Line distance × Group	0.671	.515	.025
Vision of the hands × Position of the static hand × Line distance × Which hand is used	0.068	.934	.003
Vision of the hands × Position of the static hand × Line distance × Group × Which hand is used	1.101	.340	.041
Workspace × Vision of the hands × Position of the static hand × Line distance	0.710	.497	.027
Workspace × Vision of the hands × Position of the static hand × Line distance × Group	0.512	.602	.019
Workspace × Vision of the hands × Position of the static hand × Line distance × Which hand is used	0.001	.999	.000
Workspace × Vision of the hands × Position of the static hand × Line distance × Group × Which hand is used	1.951	.152	.070
